# A Study on the Regioselective Acetylation of Flavonoid Aglycons Catalyzed by Immobilized Lipases

**DOI:** 10.3390/biom14080897

**Published:** 2024-07-24

**Authors:** Angelos Papanikolaou, Alexandra V. Chatzikonstantinou, Renia Fotiadou, Aliki Tsakni, Dimitra Houhoula, Angeliki C. Polydera, Ioannis V. Pavlidis, Haralambos Stamatis

**Affiliations:** 1 Laboratory of Biotechnology, Department of Biological Applications and Technology, University of Ioannina, 45110 Ioannina, Greece; aggelos_pap93@outlook.com (A.P.); renia.fotiadou@gmail.com (R.F.); apolyder@uoi.gr (A.C.P.); 2 Department of Food Science and Technology, University of West Attica, 12243 Athens, Greece; aliki_tsak@yahoo.gr (A.T.); dhouhoula@uniwa.gr (D.H.); 3 Department of Chemistry, University of Crete, Voutes University Campus, 70013 Heraklion, Greece; ipavlidis@uoc.gr

**Keywords:** flavonoids, acetylation, lipase, immobilization, biocatalysis, molecular docking, antimicrobial

## Abstract

This study aimed to explore the capacity of immobilized lipases on the acetylation of six aglycon flavonoids, namely myricetin, quercetin, luteolin, naringenin, fisetin and morin. For this purpose, lipase B from *Candida antarctica* (CaLB) and lipase from *Thermomyces lanuginosus* (TLL) were immobilized onto the surface of ZnOFe nanoparticles derived from an aqueous olive leaf extract. Various factors affecting the conversion of substrates and the formation of monoesterified and diesterified products, such as the amount of biocatalyst and the molar ratio of the substrates and reaction solvents were investigated. Both CaLB and TLL-ZnOFe achieved 100% conversion yield of naringenin to naringenin acetate after 72 h of reaction time, while TLL-ZnOFe achieved higher conversion yields of quercetin, morin and fisetin (73, 85 and 72% respectively). Notably, CaLB-ZnOFe displayed significantly lower conversion yields for morin compared with TLL-ZnOFe. Molecular docking analysis was used to elucidate this discrepancy, and it was revealed that the position of the hydroxyl groups of the B ring on morin introduced hindrances on the active site of CaLB. Finally, selected flavonoid esters showed significantly higher antimicrobial activity compared with the original compound. This work indicated that these lipase-based nanobiocatalysts can be successfully applied to produce lipophilic derivatives of aglycon flavonoids with improved antimicrobial activity.

## 1. Introduction

**Figure 1:**
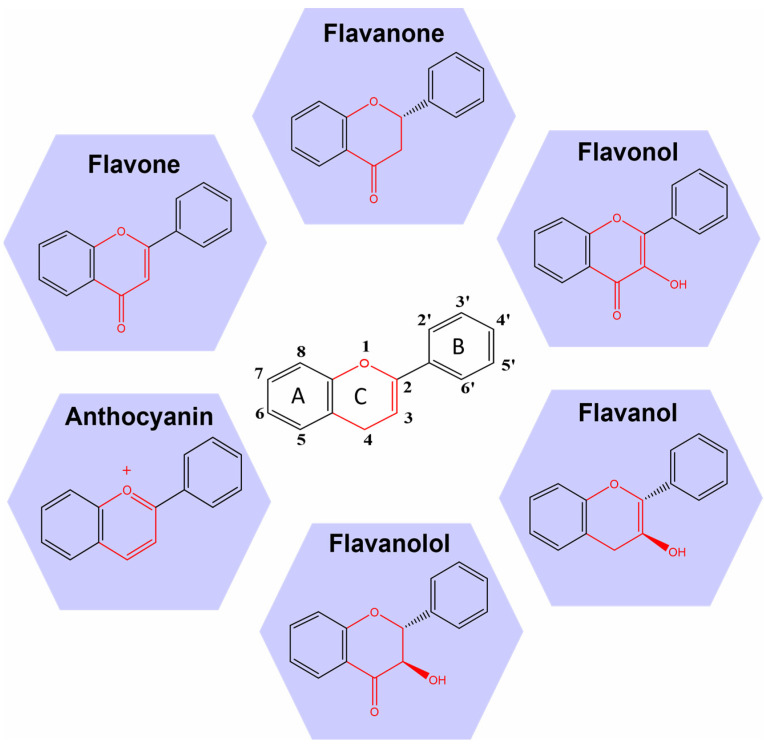
The core structure of flavonoids and the various classes. Modifications of the C ring (red) highlight the different subclasses of flavonoids.

Flavonoids constitute a major group of secondary polyphenolic metabolites that naturally occur in the plant kingdom. They represent one of the most structurally versatile compounds, accounting for more than 9000 reported in the literature [1]. The reason for their versatility lies in their core chemical structure, which consists of a fifteen-carbon skeleton, two benzene rings (A and B), and a heterocyclic ring (ring C) (Figure 1). Their structure allows for a large number of substitutions that lead to several subcategories of flavonoids such as flavonols, flavones, flavanones and many more, each of them displaying different biological activities [2,3]. Researchers have already demonstrated that their biological activities towards plants and humans, such as antioxidative, anti-inflammatory and antibacterial activity, are associated with their structural variation [4].

Flavonoids mainly occur in a glycosylated form and, due to their hydrophilic nature, they exert low stability and solubility in various lipophilic systems. Additionally, the numerous hydroxyl groups on their structure provide weak acidic properties and, as a result, their incorporation into various fields, such as pharmaceutical and food industries, is limited [5]. For this reason, researchers have long been experimenting with structural modifications of flavonoids in order to create compounds with enhanced properties and biological activities. There are many substituent groups on flavonoids, such as *O*-methylation, glycosylation, sulfation and acylation. The type of substitution mainly refers to the end product and application of the synthesized compounds [5]. The most common and widely used approach is the acylation of hydroxyl groups of flavonoids through esterification/transesterification reactions [2,6,7,8]. The nature of the acyl donor constitutes a crucial step for the modification of these compounds, since it has been proven that it influences the biological and physicochemical properties of the synthesized esters [9,10]. Acylating agents have been exploited for the synthesis of ester analogs, such as aromatic and aliphatic compounds, although the latter are the most popular ones due to their increased solubility, stability and applicability in various lipophilic systems [10].

Two of the main strategies involved in the synthesis of flavonoid esters refer to chemical and biocatalytic synthesis [2]. However, the use of specific enzymes is generally favored over the chemical approach, since the latter comprises multiple steps, such as the need to protect specific reactive hydroxyl groups on the molecule of interest to achieve the desired ester [2]. Additionally, synthetic chemical reactions are usually performed in harsh conditions and solvents which have an adverse impact on the environment while also resulting in products of low quality that hinder their applications in commercialization [11]. On the other hand, enzymatic modification of flavonoids is conducted under milder reaction conditions, which do not affect the structure of the final product and exhibits high regioselectivity, and thus the formation of byproducts, such as isomers, is minimized or non-existent [12]. Such biocatalytic approaches for the synthesis of bioactive molecules are generally considered to be green processes for application in various industrial sectors, including the chemical, pharmaceutical and food industries [11].

Lipases have been extensively incorporated in the industrial synthesis of various products, including fine chemicals [13], cosmetics [14] and pharmaceuticals [15]. These enzymes can catalyze a wide range of reactions, depending on the medium used, and have the ability to differentiate between multiple substrates [16,17]. The pivotal factor of their industrial success was the breakthrough of the immobilization that maintained their activity while bolstering their stability. This advancement enabled the reuse of biocatalysts which, in turn, led to a decrease in the overall cost of their application in various processes [18]. Current research suggests the immobilization of enzymes on nanosized materials to address the limitations of conventional chemical techniques [19,20]. Plant-derived nanomaterials have been proven to be an excellent support matrix for the immobilization of enzymes. More specifically, green hybrid zinc oxide-iron oxide (ZnOFe) nanoparticles were successfully synthesized from an aqueous olive leaf extract and utilized as support matrices for lipases. These nanobiocatalysts were applied to various aqueous and non-aqueous solvents for the synthesis of fatty acid esters [21], paving the way to a more sustainable and eco-friendly approach to commercial biocatalysis.

Lipase-mediated production of flavonoid esters has garnered a substantial amount of interest in the last couple of decades [7,12,22,23]. Enzymatic acylation is commonly carried out with flavonoid glycosides rather than aglycones due to the specific targeting of hydroxyl groups on the sugar moiety. However, acylating aglycone flavonoids is more challenging, mainly because the absence of a sugar moiety enhances the lipophilicity of the molecule, and the acylation can mask the reactive hydroxyl groups of the B-ring [7,24]. Lipase B from *Candida antarctica* (CaLB) is widely used in anhydrous conditions for the transesterification of glycosylated and aglycon flavonoids with various acyl donors [2,6,7,12,25]. In these studies, the most frequent solvents used for the acylation of flavonoids were 2-methyl-2-butanol and acetone, since these solvents present low toxicity and can lead to high conversion yields [9]. Additionally, studies performed on the acetylation of aglycon flavonoids with CaLB have been shown to acetylate the B-ring, exhibiting high regioselective patterns [22,25]. However, in the aforementioned studies, only commercial enzymes were applied for the synthesis of aglycon flavonoids. Additionally, few reports have utilized computer-based modeling methodologies to understand the mechanism and selectivity of enzyme-catalyzed reactions [26,27].

In the present work, two microbial lipases, *Candida antarctica* (CaLB) and *Thermomyces lanuginosus* (TLL), were immobilized on magnetic ZnOFe nanoparticles and were used to catalyze the regioselective acetylation of various flavones and flavonols. Additionally, some key parameters that affect the yield and the regioselectivity of the enzymatic acetylations, such as the concentration of enzymes, the molar ratio between the flavonoid and acylating agent, and the reaction solvent in the medium, were evaluated. The aglycone flavonoids under examination encompassed myricetin, quercetin, naringenin, morin, fisetin and luteolin, three of which (morin, fisetin and luteolin) have never been modified by enzymatic means before. Furthermore, computational methods, such as molecular docking, were applied to investigate, on a molecular level, the binding modes within the catalytic cavity of CaLB and TLL, as an attempt to elucidate the potential distinctions in regioselective acetylation. Finally, the acetyl derivatives of morin, fisetin and luteolin thus produced were examined for their in vitro antimicrobial activity against Gram-negative and Gram-positive bacteria.

## 2. Materials and Methods

### 2.1. Materials

Myricetin, quercetin, luteolin and fisetin (90%) were purchased from Carbosynth (Compton, Berkshire, UK); naringenin (≥95%) and vinyl acetate (>99%) were purchased from Sigma-Aldrich (St. Louis, MO, USA). Lipase B from *Candida antarctica* and lipase from *Thermomyces lanuginosus* were kindly provided by Novozymes A/S (Bagsværd, Denmark) and were used without further purification. Acetonitrile, acetone, 2-methyl-2-butanol and methyl tert-butyl ether (HPLC grade) were purchased from Fisher Scientific Corporation (Loughborough, UK); dimethyl sulfoxide-d_6_ (DMSO-d_6_) (>99%) was purchased from Deutero (Kastellaun, Germany). All other analytical grade reagents were purchased from Sigma-Aldrich (St. Louis, MO, USA).

### 2.2. Methods

#### 2.2.1. Immobilization of Lipase B from *Candida antarctica* and Lipase from *Thermomyces lanuginosus* on ZnOFe Nanoparticles (NPs)

Lipase B from *Candida antarctica* (CaLB, 57.2 U/mg) and *Thermomyces lanuginosus* (TLL, 64.4 U/mg) were immobilized on the surface of ZnOFe nanoparticles (NPs) that were synthesized from an aqueous extract of *Olea europaea* leaves. The synthesis and characterization of these nanoparticles were previously reported by Fotiadou et al. [21] and were applied for the immobilization of lipase from *Thermomyces lanuginosus*. In brief, 2 mg of ZnOFe NPs were dispersed in 2 mL of a phosphate buffer (50 mM, pH 7.5) and sonicated for 20 min. Next, different concentrations of soluble CaLB and TLL (0.5–10 mg mL ^−1^) were added to the solution and incubated for 1 h at 30 °C under constant stirring. The nanobiocatalyst was recovered by applying a magnet (grade N42) and was rinsed thrice in a phosphate buffer (50 mM, pH 7.5). Finally, it was placed into a speed-vacuum concentrator to dry and was stored at 4 °C for further use. The optimal concentration used for this study was 10 mg mL^−1^ for both cases in terms of the enzyme activity of the immobilized nanobiocatalyst, with a specific activity of 5.8 U/mg nanobiocatalyst and 6.7 U/mg nanobiocatalyst for CaLB-ZnOFe and TLL-ZnOFe, respectively (Table S1). In this study, one unit of enzymatic activity (U) was defined as the amount of lipase that produced 1 μmol of *p*-NP per minute per mL of reaction at 40 °C. The immobilization yield was calculated as described previously [21], and the protein content per mg of nanobiocatalyst was found to be 1.8 mg and 1.6 mg in the case of CaLB and TLL, respectively.

#### 2.2.2. Enzymatic Transesterification Reactions

All enzymatic transesterification reactions of aglycon flavonoids were conducted in screw-capped flasks with a working volume of 1 mL. The reactions proceeded for 72 h under a constant stirring speed (150 rpm) using a shaking incubator at 50 °C. The solvents that were used in the reactions were all dried for at least 48 h before use with the employment of molecular sieves. The concentrations of nanobiocatalysts, as well as the molar ratios of flavonoid:acyl donor, were investigated and are given in the next section. Control reactions were performed without the nanobiocatalyst, and no formation of products was detected from the acetylation reaction of flavonoids.

#### 2.2.3. Optimization of Reaction Conditions

**Figure 2:**
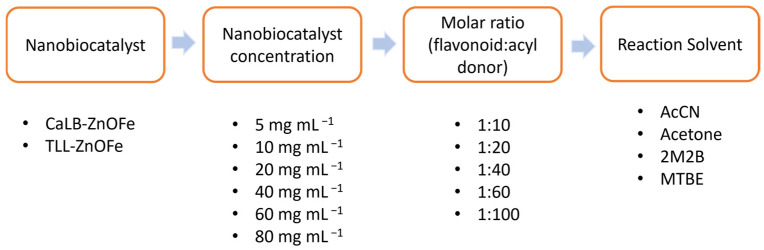
Optimization procedure for the synthesis of acetylated flavonoids.

To investigate the optimal conditions for the enzyme-mediated modification of flavonoids, the following parameters were tested in this order: the concentration of nanobiocatalysts, the molar ratio (flavonoid:acyl donor) and the reaction solvent (Figure 2).

The conjunction of myricetin with vinyl acetate was used as the reaction model. First, an experiment was conducted to investigate the optimal concentration of nanobiocatalysts. Various concentrations were used, ranging from 5 mg mL^−1^ to 80 mg mL^−1^. The molar ratio of myricetin:vinyl acetate was 1:40, the reaction temperature was set at 50 °C, and the solvent used was methyl tert-butyl ether (MTBE).

To investigate the effect of the molar ratio of myricetin:vinyl acetate, various ratios were used, ranging from1:10 to 1:100. The reaction was carried out with the optimal concentration of nanobiocatalysts, MTBE as the reaction solvent, and the temperature was set to 50 °C.

Finally, the effect of various organic solvents, namely acetonitrile (AcCN), acetone, tert-amyl alcohol (2M2B) and MTBE, on the efficiency of the enzymatic acetylation was investigated. The experiments were carried out with the optimal concentration of enzymes and molar ratio. Additionally, two distinct classes of flavonoids, namely myricetin (flavonol) and luteolin (flavone), were used as model substrates.

In the end, with the optimal experimental conditions, the enzymatic reactions were conducted with quercetin, fisetin, morin and luteolin (including myricetin and luteolin) to create a comparative profile for both nanobiocatalysts (CaLB-ZnOFe and TLL-ZnOFe). All experiments were performed in triplicate.

#### 2.2.4. Quantification, Purification and Structural Characterization of the Modified Flavonoid Esters

##### High-Performance Liquid Chromatography Analysis

The quantitative and qualitative analysis of the flavonoids and their ester analogs was performed by high-performance liquid chromatography (HPLC) with a Kinetex EVO C18 reversed-phase column (4.6 mm diameter × 250 mm length column, 5 μm particle size) and a diode array UV detector. The column’s temperature was set to 30 °C.

The mobile phase consisted of acetonitrile (A) and water (B, with 0.1% acetic acid) with a gradient elution of 10–90% A to 100–0% A at 0–30 min with a flow rate of 1 mL min^−1^. The following wavelengths were used for the detection of each flavonoid and their respective flavonoid esters using a PDA detector: myricetin, 370 nm; luteolin, 348 nm; naringenin, 288 nm; quercetin, 370 nm; morin, 254 nm; and fisetin, 360 nm. The conversion yield was defined as the amount of product formed (modified flavonoid), and the amount of the modified flavonoid was determined by Equation (1).

(1) $$Conversion\;yield\;\left(\%\right)=\frac{Area\;of\;Product}{Area\;of\;substrate+Area\;of\;all\;Products}\times 100$$

From the reaction, aliquots of 20 μL were withdrawn after 24, 48 and 72 h, and were separated from the enzyme with a magnet. The reaction aliquots were diluted 1:16 in methanol and filtered through a nylon-type filter with a 0.22 μm pore diameter before being injected for analysis. To identify unreacted flavonoids in the reaction mixture, the retention times of the pure flavonoid substrates were compared with those of the peaks in the reaction mixture. Newly formed peaks which did not match the retention times of the pure substrates were identified as reaction products and were further identified by other analytical techniques.

The acetylated flavonoid mixtures were purified using a semi-prep Luna C18 column (250 × 10 mm, 10 μm). The mobile phase consisted of acetonitrile (A) and water (B, with 0.1% acetic acid), with a gradient elution of 10–90% A to 100–0% A at 0–30 min with a flow rate of 5 mL min^−1^. The fractions were collected and concentrated under a vacuum in a rotavapor (Buchi Rotavapor R-114, Büchi Labortechnik AG, Flawil, Switzerland). Afterwards, they were subjected to lyophilization and then used for structural identification and antimicrobial analysis.

##### Mass Spectrometry Analysis

Mass spectrometry analysis was conducted with an Advion mass spectrometer system, featuring an atmospheric pressure chemical ionization (APCI) interface, following the protocol mentioned before [28,29] with slight modifications. The mass analyzer was a single quadrupole. The quadrupole consisted of four parallel rods that used electric fields to filter ions based on the *m*/*z* ratio. The type of detector which was used was an electron amplifier. The conditions used for mass spectrometry for the positive source of APCI were nebulizing gas (N_2_) with a flow rate of 1.5 mL min^−1^, a capillary voltage of 180 V, a source voltage offset of 25 V, a source voltage span of 20 V, a source gas temperature of 350 °C, a capillary temperature of 200 °C and an APCI corona discharge of 5 μA. A volume of 10 μL of the sample was directly injected. Samples were dissolved in methanol. For full-scan MS analyses, the spectra were recorded in the positive mode in the range of *m*/*z* 100–1000. All spectra are provided in the Supporting Information.

##### Nuclear Magnetic Resonance Analysis

The quantitative analyses and characterization of the acetylation products were determined by ^1^H-^13^C HSQC-HMBC NMR experiments. After the completion of the reaction, the immobilized enzyme was removed by applying a magnetic field, the solvent was removed with a rotary evaporator, and the crude products were then redissolved in 500 μL of DMSO-d*_6_* and transferred to a 5 mm NMR tube. All NMR experiments were performed on a Bruker 500 MHz AV spectrometer equipped with a cryoprobe (Bruker BioSpin, Rheinstetten, Germany) at 273 K. The NMR system was controlled by the software TopSpin 4.0. The pulse sequences for ^1^H-^13^C HSQC and ^1^H-^13^C HMBC were standard Bruker library sequences, acquired with 2 K data points over a 14 ppm spectral width. The ^1^H and ^13^C NMR chemical shifts (δ, ppm) of the compounds are given in the Supporting Information.

#### 2.2.5. Assay of Antibacterial Activity

The antibacterial activity of selected flavonoids, namely fisetin, luteolin and morin, before and after the enzymatic acetylation, was estimated according to Chatzikonstantinou et al. [30] against *Escherichia coli* BL21 (*E*. *coli*) and *Corynebacterium glutamicum* ATCC 21253 (*C. glutamicum*). In brief, bacterial cells were grown overnight in an LB broth medium under shaking conditions at 37 °C. After the incubation, the bacterial cells were added to a 0.9% *w/v* NaCl solution (with a concentration of ~10^8^ CFU mL^−1^) and were allowed to interact overnight (~16 h) with various concentrations of the tested compounds, ranging from 10–200 μg mL^−1^, at 37 °C under shaking conditions. Finally, an appropriate amount of the samples was transferred to a 96-well microplate containing LB broth so that the final concentration of the bacterial cells was ~10^7^ CFU mL^−1^. Afterwards, the plates were incubated for 8 h under shaking at 37 °C, and their absorbance was monitored at 600 nm a 1-h intervals to determine their growth curves. The IC_50_ values, which defined the concentration that inhibited the growth of the bacterial population by 50%, were determined by fitting a non-linear dose-dependent four-parameter curve. All measurements were performed in triplicate.

#### 2.2.6. In Silico Analysis

All in silico experiments were performed using the YASARA modeling suite (YASARA structure v 21.8), and images were generated using PyMOL v0.99.

##### Preparation of the Protein Structures—Construction of the Acyl-Enzyme

The coordinates of the 3D protein structures for both CaLB and TLL were downloaded from RCSB (https://www.rcsb.org/ (accessed on 25 November 2023)) with the PDB ID codes 1TCA and 6OR3, respectively. The crystal structure of TLL (6OR3) is an acyl intermediate of palmitic acid which is covalently bound to the catalytic serine (Ser146) residue. To properly optimize the structure, the aliphatic chain of the palmitic acid was removed, while the acetate on the hydroxyl side chain of Ser146 was kept as it was. The orientation of the acetate helped provide experimental evidence of the interactions between the acyl moiety and the residues that make up the oxyanion hole (Ser83 and Leu147). The NAG ligand was removed as well as all water molecules from the structure. Similarly, the hydroxyl side chain of CaLB’s catalytic serine (Ser105) was replaced by acetate to mimic the acyl-enzyme complex. All water molecules and ligands were removed as well. Missing hydrogen atoms were added, and their hydrogen bonding network was optimized via the program’s utility [31]. Both the catalytic Asp and His residues were deprotonated (Asp187 and His224 for CaLB, and Asp201 and His258 for TLL). The resulting structures underwent an energy minimization procedure to remove bumps and correct their covalent geometry. In order to preserve the crystalline organization of the protein atoms, constraints and restraints were introduced that were progressively removed. YASARA2 Force Field was used, since it has been optimized for the refinement of proteins and contains knowledge-based dihedral and interaction potentials [32]. An 8 Å force cutoff and the particle mesh Ewald algorithm [33] to treat long-range electrostatic interactions were utilized. After the removal of conformational stress by a short minimization using the steepest descent method, the procedure continued by simulated annealing until convergence was reached. Both structures during the procedure were solvated in water in a 10 Å periodic simulation cell. The refined structures were then used as a starting point for molecular docking.

##### Ligand Preparation

The ligands were constructed using YASARA’s molecule builder. Afterwards, they were subjected to an energy minimization procedure using YASARA2 Force Field. This step was performed to optimize the geometry of the tested compounds while checking the bond orders, the number of bonds and the valence.

##### Flexible Docking Protocol

Molecular docking was performed utilizing YASARA’s embedded macro. Both the receptor’s side chains and ligands were kept flexible. The temperature, which affected only the receptor’s flexibility, was set to 50 °C to represent the temperature at which the enzymatic reactions took place. The docking experiments were run with AutoDock Vina. For CaLB, a simulation cell that extended for 14, 11 and 14 Å in each axis (*x*, *y* and *z*) around the Oγ atom of the catalytic acyl-serine (Ser146), and for 13, 11 and 13 Å (*x*, *y* and *z*) cell around Ser105 for TLL were built in order to envelope the active site’s cavity. During the procedure, 25 docking runs were executed for each protein assembly (5 in total) resulting in a total of 125 possible ligand–receptor binding conformations. The resulting different ligand conformations clustered when the ligand’s RMSD was lower than 4 Å.

##### Analysis of the Docking Results

The mechanism of a lipase-catalyzed acylation reaction involves the participation of five residues within the active site of CaLB and TLL. In CaLB, the residues Ser105, Asp187 and His224 together form the catalytic triad, while Thr40 and Gln106 constitute the oxyanion hole residues. Similarly, the catalytic triad of TLL is composed of Ser146, Asp201 and His258, while Ser83 and Leu147 form the oxyanion hole. On the basis of the structural insights, the complexes that were generated from docking simulations were analyzed through a set of parameters as reported previously [34]:

- The stability of the hydrogen bonding interactions with the oxyanion hole;
- The ace:carbon atom from the acyl-serine and the substrate’s oxygen atom from the hydroxyl group to be acetylated must be at a distance of less than 4 Å;
- The acidic proton of the hydroxyl group to be acetylated must be at a distance of 4 Å or lower from the catalytic histidine.

##### Statistical Analysis

The experiments were carried out in triplicate independently, and the results are presented as the mean ± standard deviation. The SD is indicated as error bars in the plots. Student’s *t*-test, one-way ANOVA and Tukey’s multiple comparison test were carried out using IBM SPSS statistics (version 21, Armonk, NY, USA: IBM Corp.).

## 3. Results and Discussion

### 3.1. Optimization of the Reaction Conditions

#### 3.1.1. Effect of the Concentration of Enzymes on the Conversion of Myricetin

**Scheme 1:**
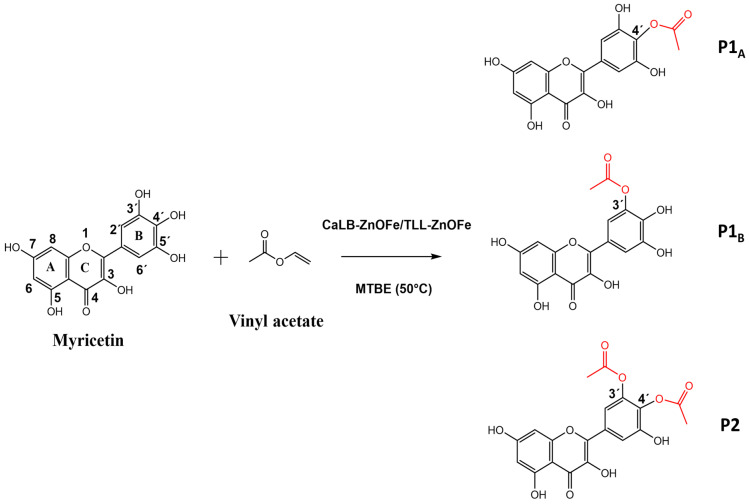
Acetylation reaction of myricetin by CaLB-ZnOFe and TLL-ZnOFe. The acetylated groups are show in red.

The concentration of enzymes affects the reaction rate and thus the conversion of the acetylation reaction. To investigate the effect of enzyme concentrationon the acetylation of flavonoids, myricetin was used as a model reaction (Scheme 1), since it represents a compound with the highest number of hydroxyl groups among the flavonoids used in this study, providing more potentialacetylation sites. A series of reactions were performed by incorporating the immobilized lipases, CaLB-ZnOFe and TLL-ZnOFe, at different concentrations ranging from 5 mg mL^−1^ to 80 mg mL^−1^. The reactions were performed in MTBE and the molar ratio of flavonoid:vinyl acetate was 1:40.

The reactions were monitored through HPLC analysis, and the retention times of the unreacted myricetin and its products were found (Figure S1). According to the chromatogram, Peak S1 was the unreacted myricetin, while Peaks P1 and P2 were the products of the reaction. To structurally identify the products, the samples were analyzed by ACPI-MS. The depicted molecular masses on the spectra identified P1 as monoacetate (with a molecular ion *m*/*z* of 361) and P2 as diacetate (with a molecular ion *m*/*z* of 403) (Figure S7). Further structural identification and characterization of the esters by NMR spectroscopy (^1^H and ^13^C NMR) showed that the products of P1 were two monoesters, myricetin 4′-acetate and myricetin 3′-acetate, henceforth referred to as P1_A-B_, and P2 was identified as 3′,4′-diacetate myricetin ester.

**Figure 3:**
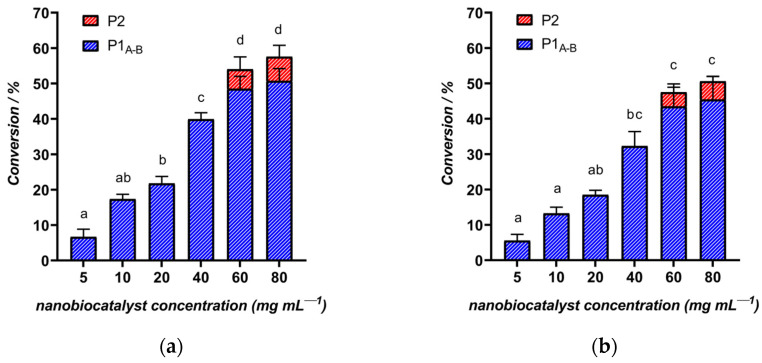
Conversion yield (%) of myricetin with (**a**) CaLB-ZnOFe and (**b**) TLL-ZnOFe, influenced by the concentration of nanobiocatalysts (mg mL^−1^) in MTBE after 72 h of reaction time. The molar ratio of myricetin:vinyl acetate was 1:40 for both cases; the reaction temperature was set to 50 °C. Means with the same letter are not statistically significant according to Tukey’s multicomparison test (at *p* > 0.05).

Figure 3a,b illustrates the effect of the concentration of nanobiocatalysts on the acetylation of myricetin. The increase in the concentration of nanobiocatalysts in the reaction mixture led to an increase in the overall conversion yield of myricetin. However, further increase in the amount of nanobiocatalysts above 60 mg mL^−1^ did not significantly affect the conversion of myricetin. It was previously reported that an excessive concentration of enzyme might negatively impact the conversion due to the mass transfer limitations of the products and substrates [35,36]. The major product of the reaction mixture, in both cases, was the 4′- and 3′-acetate ester of myricetin (P1_A-B_), while the diacetate (P2) was the minor product (Figure 3). The formation of the produced diester was low, but this could be attributed to many factors, such as the reaction time [37]. Further increases in the reaction time might have a greater impact for the formation of a diester than the concentration of enzymes. For these reasons, the optimal concentration of nanobiocatalysts was set to 60 mg mL^−1^.

#### 3.1.2. Effect of the Molar Ratio on the Conversion of Myricetin

**Figure 4:**
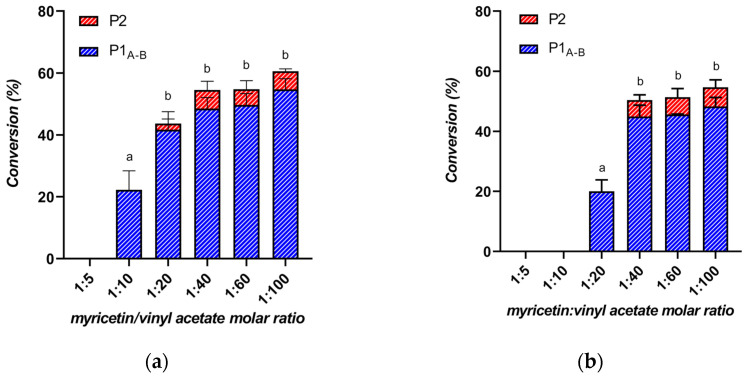
Conversion yield (%) of myricetin with (**a**) CaLB-ZnOFe and (**b**) TLL-ZnOFe influenced by the molar ratio between myricetin and vinyl acetate after 72 h of reaction time in MTBE. The optimal concentration of the nanobiocatalyst was used (60 mg mL^−1^), and the reaction temperature was set to 50 °C. Means with the same letter are not statistically significant according to Tukey’s multicomparison test (at *p* > 0.05).

The molar ratio of the substrates (flavonoid:vinyl acetate) can impact the reaction rate and, consequently, the conversion yield of the enzymatic acetylation reactions [9]. In the present work, we investigated the effect of different molar ratios of myricetin:vinyl acetate on the conversion yield and regiosectivity of the reaction. As illustrated in Figure 4a,b, an increase in the molar ratio leads to an increase in the conversion yield of myricetin. In the case of CaLB-ZnOFe, an increase in the molar ratio from 1:5 to 1:40 drastically increased the conversion yield of myricetin. However, the subsequent increase in the concentration of vinyl acetate only resulted in a marginal increase in the production of esters. A similar outcome regarding TLL-ZnOFe was observed. The production of monoacetate myricetin esters (P1_A-B_) was evident at a substrate ratio of 1:20, and a notable increase in the reaction yield was observed with an increase in vinyl acetate. Further increases in the acyl donor did not significantly affect the formation of myricetin ester. Our findings from this experiment are supported by similar works in which they utilized immobilized lipases for the modification of aglycon and glycosylated flavonoids [35,36,38]. In these works, they showed a positive correlation between an increase in the target substrate’s conversion yield and the increase in the acyl donor. The reaction rate and conversion of esters reached a saturation point in which further increases in the molar ratio did not affect the reaction. This was due to the excess of the acyl donor, which had no further effect on the thermodynamic shift in the reaction’s equilibrium towards transesterification.

Furthermore, only a few studies have considered the effect of the molar ratio on the enzyme’s regioselectivity. In a work conducted by Chebil et al. [39], an immobilized lipase (PCL) was utilized for the acetylation of quercetin. They observed that with an excess of the acyl donor (vinyl acetate), the proportion of monoester that formed decreased, while the formation of di- and triester products was favored. In our work, the results showed that an increase in the molar ratio had a minor effect on the formation of a diester (P2), while no decrease in the formation of monoesters was observed (Figure 4). The discrepancy between these results might be attributed to the different biocatalysts that were used in the two studies. Indeed, Zhu et al. [38] observed that an excess of the acyl donor for the acylation of dihydromyricetin had a minor influence on the reaction’s regioselectivity, utilizing TL IM as a biocatalyst.

Therefore, a molar ratio of 1:40 was considered to be the optimal molar ratio for both cases in order to keep the same parameters between the two nanobiocatalytic systems.

#### 3.1.3. Effect of the Solvent Type on the Conversion of Myricetin and Luteolin

It is well known that the performance and the selectivity of an enzymatic reaction are significantly affected by the solvents used in the reaction system [8,35,36,39,40]. Moreover, the solubility of substrates and products is expected to affect the reaction’s progress, and it is important to choose a reaction medium in which both flavonoid and acyl donor are adequately soluble [41]. In our work, the effect of the solvents on the progress and the selectivity of the enzymatic acetylation of flavonols and flavones was investigated using the acetylation of luteolin and myricetin as model reactions.

**Scheme 2:**
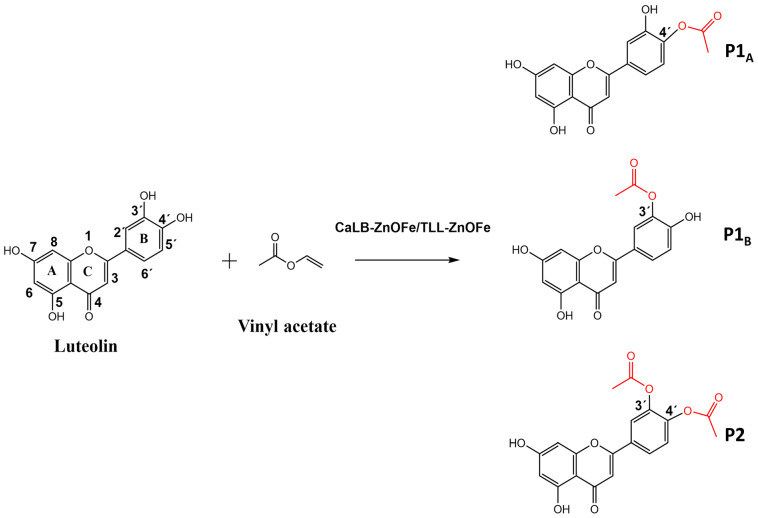
The acetylation reaction of luteolin by CaLB-ZnOFe and TLL-ZnOFe. The acetylated groups are shown in red.

In a similar manner, as described before for the acetylation of myricetin, the enzymatic acetylation of luteolin led to the formation two monoesters (P1_A-B_) and a diester (P2), as indicated by MS (Scheme 2, Figures S3 and S7). Further structural identification and characterization of the esters by NMR spectroscopy (^1^H and ^13^C NMR) showed that the products of P1_A-B_ were two mono-substituted products, namely luteolin 4′-acetate and 3′-acetate, and those of P2 were luteolin 3′,4′-diacetate.

Table 1 and Table 2 display the conversion yields of myricetin and luteolin, respectively. The higher conversion yield for both enzymes tested was observed with MTBE, while in the other solvents, the conversion was significantly lower. Additionally, the absence of the production of diesters in the case of 2M2B has been discussed in a previous study with CaLB [42]. Moreover, the formation of diesters was only observed in the case of MTBE. It was proposed that organic solvents with high polarity, such as acetonitrile and acetone, can destabilize the water layer on the lipases’ surface, which negatively affects their catalytic activity [43,44]. Furthermore, polar solvents can interact with the active site and destabilize the hydrogen bonding network, which, in turn, significantly affects the enzyme’s activity [45]. In a similar study by Zhu et al. [38], they found that MTBE was less destructive for the water layer on the lipase’s surface (TLL), and the acylation yield of dihydromyricetin was much higher compared with other solvents. Fotiadou et al. showed that TLL-ZnOFe showed better activity in non-polar solvents [46], further supporting the findings of this work.

Additionally, the different conversion yields of myricetin and luteolin could also be related to their solubility in the reaction medium. In our study, both compounds displayed poor solubility in MTBE compared with the other solvents with the highest conversion yields. The lower solubility of substrates in a reaction medium with higher conversion yields or reaction rates was also supported in other works and it is partly related to the relative solvation of the substrate which is bound to the enzyme and the substrate in the solution [47,48]. When a substrate is more soluble in a particular solvent, its effective concentration relative to the enzyme decreases, resulting in the substrate’s reduced activity and availability for enzyme binding and reaction [47,48].

According to these results, MTBE is the optimal solvent for the process of acetylation of myricetin and luteolin.

The final optimal parameters for the acetylation of aglycon flavonoids were determined to be a concentration of nanobiocatalysts of 60 mg mL^−1^, a molar ratio of 1:40 (substrate:acyl donor) and MTBE as the reaction solvent.

#### 3.1.4. Acetylation of Various Aglycon Flavonoids with Optimized Parameters

**Figure 5:**
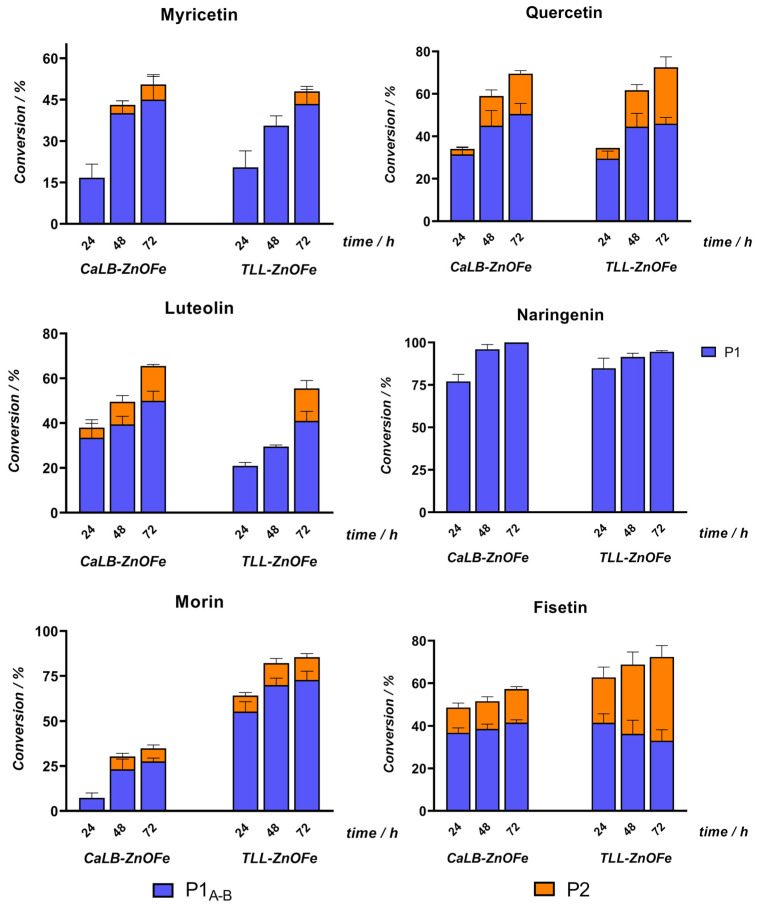
Conversion yield (%) of optimized acetylation reactions of various aglycon flavonoids by CaLB-ZnOFe and TLL-ZnOFe. Conversion yields are shown by red letters above the bars after sampling at 72 h.

The ability of CaLB-ZnOFe or TLL-ZnOFe to catalyze the acetylation of various dietary aglycon flavonoids, which display numerous biological activities, was also investigated. As can be seen in Figure 5, the conversion yield depended on the aglycon flavonoid and the nanobiocatalyst used. The formation of mono- and diesters was observed for the acetylation of all flavonoids tested except for naringenin, where only a monoester was detected (Figure 5). Naringenin was 100% converted to 4′-naringenin acetate by both CaLB-ZnOFe and TLL-ZnOFe after 48 h of incubation, which was in accordance with that reported elsewhere using the commercial lipase B from *Candida antarctica* (Novozyme 435), albeit with a lower conversion yield compared with both CaLB-ZnOFe and TLL-ZnOFe conjugates [22]. Moreover, the high regioselective acetylation of naringenin observed here contrasts with that reported by Chebil et al., [39] where lipase from *Pseudomonas cepaciae* (PCL) produced naringenin 4′-acetate and naringenin 7,4′-diacetate esters, indicating the effect of the biocatalyst used on the regioselectivity of the reaction. Additionally, from Figure 5, it can be seen that the amount of the flavonoids’ diesters produced was associated with the conversion of aglycon flavonoids and the formation of the monoacetylated derivatives, which is in accordance with that reported elsewhere for the enzymatic acylation of various glucosylated flavonoids [48].

In the work conducted by Chebil et al., quercetin was successfully acetylated by PCL but exhibited low regioselectivity, which resulted in the formation of three products, 3′-acetate, 3′,4′-diacetate and 7,3′,4′-triacetate esters [39]. Both nanobiocatalysts used in the present work (CaLB-ZnOFe and TLL-ZnOFe) displayed higher regioselectivity, as was evident from the formation of the products (Figure 5, Table 3), which is in accordance with that reported by Kyriakou et al. [22]. The reported total conversion yield of quercetin by PCL was 96%, while in this study, it was 69% and 72% for CaLB and TLL-ZnOFe, respectively. One possible reason for the difference in the conversion yield could be attributed to the longer incubation time applied (96 h instead of 72 h). Moreover, the different lipases used can also affect the stereochemistry between the substrate and the enzyme, affecting the yield of the product [49]. Compared with the previous work [22], both CaLB-ZnOFe and TLL-ZnOFe achieved higher conversion yields of quercetin as opposed to Novozyme 435.

To the best of our knowledge, no published work exists in the literature regarding the enzymatic modification of fisetin, morin and luteolin. All three flavonoids display numerous biological activities that have been previously studied [50,51,52], and thus they present interesting targets for enzymatic modification. As can be seen from Figure 5, TLL-ZnOFe exhibited a higher affinity to fisetin due to the higher conversion yield and its ability to form high amounts of diacetate fisetin esters. In contrast, CaLB-ZnOFe displayed higher affinity to acetylate luteolin. In the case of morin, TLL-ZnOFe displayed a significantly higher conversion yield compared with CaLB-ZnOFe. This discrepancy could be attributed to the specific structure of the B-ring. Morin is the only compound in this study that has an -OH group in the 2′ position. Therefore, to gain an insight into the structural differences that might influence the affinity of morin between CaLB-ZnOFe and TLL-ZnOFe, molecular docking was used.

### 3.2. In Silico Analysis of the Acetylation of Morin and Its Ester Derivatives

Molecular docking is an effective tool for studying the interactions between enzymes and small molecules. To the best of our knowledge, only two reports exist in the literature that have utilized molecular modeling to shed light on the capacity of enzymes to catalyze the acetylation of flavonoids [26,27], and only the work of Christelle et al. [26] studied the acetylation of aglycon flavonoids. In this study, a molecular docking approach was used to investigate the binding modes of morin, along with its acetylated monoester derivative, in the binding site of CaLB and TLL to rationalize the differences observed in their affinity. The modeling proposed here considered the binding of the flavonoid on the acetylated enzymes to form the second tetrahedral intermediate. The outlined methodology was based on several stability and proximity criteria that were mentioned earlier [34]) which differentiated productive from non-productive binding conformations of the flavonoids inside the active site.

#### 3.2.1. Molecular Docking of Morin and Its Derivative on CaLB

The active site of CaLB is narrow and it is not covered with a lid, contrary to most known lipases [53]. Inside the pocket, there are mostly aliphatic residues (hydrophobic environment) and only a small slightly polar region, which consists of the side chains of Thr40, Asp134 and Gln157 near the catalytic Ser105.

**Figure 6:**
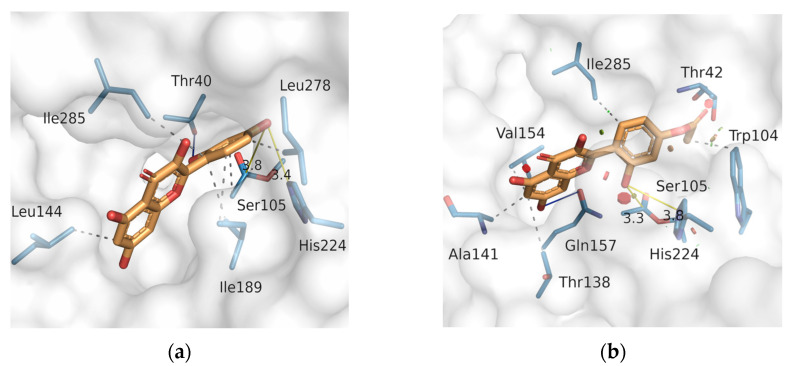
Binding mode of the catalytically active conformation of morin (**a**) into CaLB’s binding pocket. Dashed lines represent hydrophobic interactions, while the solid blue line represents hydrogen bonds. (**b**) Binding mode of acetyl-morin inside the active site of CaLB. The steric hindrances are depicted as cylinders (red cylinders indicate higher hindrances, while smaller green and orange cylinders indicate smaller steric hindrances). Distances are represented by solid yellow lines.

As a first step, morin was docked into the active site of CaLB to study the underlying interactions. The docking results showed that morin can bind productively (as per the criteria described previously [34]) inside the active site. More specifically, the B ring is oriented towards the pocket of CaLB (Figure 6a) and the 4′-OH group is positioned near the Ne2 atom of the imidazole ring of His224 (3.4 Å), facilitating the proton transfer of the hydroxyl group. However, the group is slightly further away from the carbon atom of the acyl-serine intermediate (3.8 Å). Additionally, the B ring is stabilized by the hydrophobic interaction with Leu278 and the hydrogen bond between the 2′-OH group of morin and the OH group of Thr40. The heterocyclic part of morin (ring A and C) interacts with the hydrophobic residues of the pocket, which are mainly leucines and isoleucines, and further support the catalytically active orientation (Table S3). The predictions presented here were corroborated by our experimental results, since the main product of acetylation of morin for CaLB-ZnOFe is the 4′ acetate morin.

To further complement the study and reveal the underlying interactions that lead to the formation of diacetyl-morin, we acetylated morin on the 4′-OH group (the main product from the reaction mix) and docked the derivative onto the active site of CaLB. In the results obtained, acetylated morin bound in the active site, similar to morin (Figure 6b), with slight conformation changes. More specifically, multiple hindrances were introduced, mainly between the 2′-OH group of morin and the methyl group of acyl-serine, as well as the carbonyl oxygen atom of the acyl group and the methyl group of Thr42. The position of the 2′-OH, which is unique among the flavonoids studied here, poses a challenge for CaLB to accommodate and acetylate. Coupled with the unfavored interactions between the aforementioned groups, this further indicated that CaLB exhibits a low affinity for morin and its derivatives, which was confirmed by the experimental results.

#### 3.2.2. Molecular Docking of Morin and Its Derivative on TLL

The active site of TLL seems to display slightly more polar characteristics than CaLB, probably because the active pocket is not as steep and narrow but is rather more exposed to the surface. The structure used in this study was in an open conformation, making it suitable for the docking analysis. Similarly, we followed the same procedure as in the case of CaLB to study the interactions between morin and its derivative within the active site of TLL.

**Figure 7:**
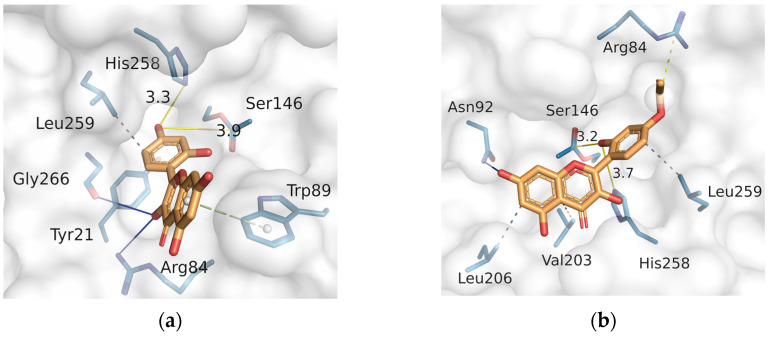
(**a**) Binding mode of the catalytically active conformation of morin into TLL’s binding pocket. Dashed lines represent hydrophobic interactions, while the solid blue line represents hydrogen bonds. The green dashed-lines between Trp89 and the A ring of morin represent the π-stacking interactions. (**b**) Binding mode of catalytically active 4′-acetyl-morin derivative into TLL’s active site. The dashed lines yellow between the acetyl group of morin and Arg84 represent interactions of the salt bridge. Distances are represented by solid yellow lines.

According to the docking results, the productive binding mode of morin shares some similarities with that of CaLB. More specifically, the B ring is oriented towards the catalytic site of TLL with the 4′-OH group in proximity to the Ne2 atom of His258 (Figure 7a). In this orientation, the B ring partakes in hydrophobic interactions between Tyr21 and Leu259, and a hydrogen bond with the catalytic His258, which facilitates proton transfer; thus, higher yields of 4′-acetate could be achieved, as was demonstrated in the experimental results. Additionally, multiple interactions helped stabilize the rest of the structure in this catalytically active conformation (Figure 7a, Table S4). Compared with CaLB, the heterocyclic ring is positioned in the solvent’s environment, which could possibly facilitate the rate of diffusion of the final product.

Similarly, we acetylated morin on the 4′-OH group to further investigate the possibility of a diacetyl morin ester. According to the docking results, mono-acetylated morin binds into the active site of TLL, with a different conformation to accommodate the acyl group (Figure 7b). More specifically, the B ring rotates so that the 2′-OH group is orientated towards the ace:carbon atom of Ser146 and above the Ne2 atom of His258. Furthermore, a hydrogen bond between the 7-OH group of the A ring and Asn92 stabilizes the core of the molecule. From the results, it is evident that the position of 2′-OH does not pose any challenge for its acetylation, since no hindrance was observed between the substrate and the neighboring residues of TLL, as opposed to CaLB. The predictions of our docking experiment aligned with the experimental findings and further supported the idea that TLL displays a higher affinity for morin and its esters compared with CaLB.

### 3.3. Antimicrobial Activity of Acetylated Flavonoids

Flavonoids’ biological activities, such as antioxidant and antimicrobial activities, to name a few, have been widely proven to be of great importance for various industries, such as food and nutraceuticals [54,55]. Many reports in the literature have shown that structural modifications of the flavonoids’ structure can affect their physicochemical and biological properties [56]. In this study, the antimicrobial activity of the aglycon flavonoids fisetin, luteolin and morin was evaluated before and after their enzymatic modification against two bacterial strains, *Escherichia coli* BL21 (*E*. *coli*) and *Corynebacterium* ATCC 21253 (*C*. *glutamicum*), which represent a Gram-negative and a Gram-positive bacterium, respectively. The selection of these flavonoids was based on the absence of literature reports regarding the antimicrobial activities of their acetyl derivatives. Since the separation and purification of the product of P1_A-B_ monoesters was not feasible, the modified compounds of fisetin and luteolin were mixtures of the two 4′- and 3′-monoacetate esters. However, in the case of morin, the product of P1 consisted solely of the 4′-acetate morin derivative.

**Figure 8:**
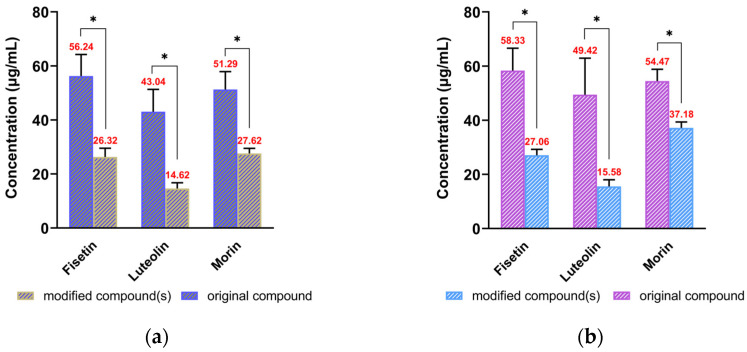
Antimicrobial activity of fisetin, luteolin, morin and their derivative esters against (**a**) *E. coli* and (**b**) *C. glutamicum*. Modified compounds represent the 3′-acetate and 4′-acetate esters in the case of fisetin and luteolin, and the 4′-acetate esters in the case of morin. Mean values are represented by red color above the error bars. Asterisks indicate statistically significant differences according to a *t*-test (*p* < 0.05).

The impact of the flavonoids and their acetylated derivatives on bacterial cell populations, expressed as IC_50_ values, which are defined as the concentration of the compound needed to reduce the growth of the bacterial population by 50%, is depicted in Figure 8a,b. Our findings revealed that the ester derivatives of the investigated flavonoids exhibited enhanced antimicrobial activity compared with the original compounds. Some recent comprehensive reviews suggested that flavonoids exert their antimicrobial effects partly by interacting and damaging the lipid bilayer of the bacteria [2,57,58,59,60]. Furthermore, these studies emphasized that the mechanism of action of flavonoids varies, depending on their structural diversity. More specifically, differences in the hydroxyl and methoxy groups’ distribution, or lack thereof, on their structure could influence their interactions with the microbial membranes [58]. In this case, the acetylation of flavonoids was proven to enhance their antimicrobial efficacy, possibly due to the stabilization of the phenolic moiety and the lipophilicity of the compound, which, in turn, influenced the interaction against the lipid bilayers of the bacteria membranes, thus inhibiting their growth [61,62].

## 4. Conclusions

This is the first report that investigated the capacity of immobilized lipases onto ZnOFe nanoparticles for the acetylation of aglycon flavonoids. In this framework, CaLB and TLL were successfully immobilized onto the surface of ZnOFe nanoparticles and applied for the acetylation of various classes of aglycon flavonoids. The optimal parameters for the reactions were investigated, and it was revealed that the choice of solvent was more conducive to the yield of flavonoid esters, while the concentration of nanobiocatalysts and the molar ratio of flavonoid:acyl donor strongly influenced the progression of the acetylation reactions. Comparative analysis between the two nanobiocatalysts revealed their high efficacy in producing flavonoid esters, selectively acetylating hydroxyl groups on the B ring. Molecular docking analysis revealed that CaLB’s lower affinity for morin was possibly due to geometric constraints within its active site. Lastly, the enhanced biological activities of the ester derivatives of fisetin, luteolin and morin could be attributed to their improved lipophilicity, potentially expanding their applicability in various industries such as food and nutraceuticals.

## Figures and Tables

**Table 1:** Conversion yield (%) of myricetin by CaLB-ZnOFe and TLL-ZnOFe with vinyl acetate as an acyl donor in various organic solvents after 72 h of reaction time. The temperature was set to 50 °C for all solvents.

Solvent	LogP	Conversion Yield, %			
		CaLB-ZnOFe		TLL-ZnOFe	
		P1	P2	P1	P2
Acetonitrile	−0.33	24.5 ± 0.7	n.d.	35.5 ± 3.5	n.d. *
Acetone	−0.24	28.5 ± 2.5	n.d.	33.8 ± 5	n.d.
Methyl-tert-butyl-ether (MTBE)	0.94	48.4 ± 4.0	6.1 ± 3.4	45.4 ± 4.4	4.5 ± 0.9
2-methyl-2-butanol (2MB2)	1.09	20 ± 3.8	n.d.	12.5 ± 3.0	n.d.

* n.d. = not detected.

**Table 2:** Conversion yield (%) of luteolin by CaLB-ZnOFe and TLL-ZnOFe with vinyl acetate as an acyl donor in various organic solvents after 72 h of reaction time. The temperature was set to 50 °C for all solvents.

Solvent	LogP	Conversion Yield (%)			
		CaLB-ZnOFe		TLL-ZnOFe	
		P1	P2	P1	P2
Acetonitrile	−0.33	6.5 ± 2.1	n.d.	17 ± 2.8	n.d. *
Acetone	−0.24	16.5 ± 3.5	n.d.	12.1 ± 3.2	n.d.
Methyl-tert-butyl-ether (MTBE)	0.94	50.8 ± 3.6	14.5 ± 3.1	35.1 ± 3.3	17.3 ± 3.9
2-methyl-2-butanol (2MB2)	1.09	5.9 ± 2.7	n.d.	4.4 ± 1.6	n.d.

* n.d. = not detected.

**Table 3:** Products formed by the enzymatic acetylation of aglycon flavonoids.

Substrate	Structure	P1_A-B_ *	P2 *
Myricetin	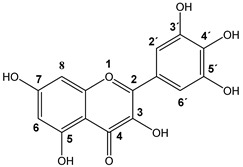	4′-acetate/3′-acetate	3′,4′-diacetate
Quercetin	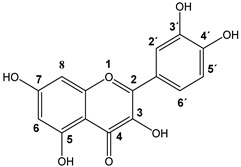	4′-acetate/3′-acetate	3′,4′-diacetate
Luteolin	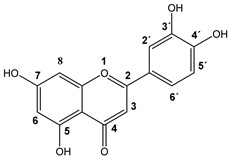	4′-acetate/3′-acetate	3′,4′-diacetate
Naringenin	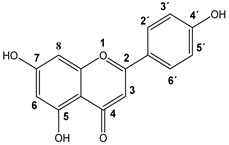	4′-acetate	n.d. **
Morin	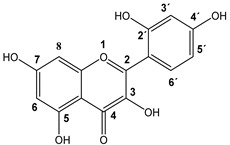	4′-acetate	2′,4′-diacetate
Fisetin	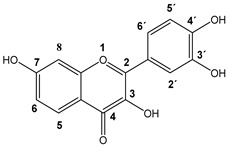	4′-acetate/3′-acetate	3′,4′-diacetate

* These results represent the products formed by both nanobiocatalysts (CaLB-ZnOFe and TLL-ZnOFe). ** n.d. = not detected.

## Data Availability

The original contributions presented in the study are included in the article/supplementary material, further inquiries can be directed to the corresponding authors.

## Supplementary Materials

The following supporting information can be downloaded at: https://www.mdpi.com/article/10.3390/biom14080897/s1, Table S1: Effect of enzyme to nanosupport mass ratio on the immobilization yield and the activity of the immobilized nanobiocatalysts. Table S2: Flavonoids and their derivatives with their expected masses (*m*/*z*). Table S3: Molecular interactions between morin and acetylated morin within the active site of CaLB. Distance interactions between carbon atoms and distance between donor and acceptor atoms (hydrogen bonds) are displayed in the 4th and 5th row respectively. Table S4: Molecular interactions between morin and acetylated morin within the active site of TLL. Distance inter-actions between carbon atoms and distance between donor and acceptor atoms (hydrogen bonds) are displayed in the 4th and 5th row respectively. Figure S1: HPLC chromatogram of the enzymatic acetylation of myricetin by TLL-ZnOFe monitored at 370 nm. The peaks at 9.57, 11.33 and 13.19 min correspond to myricetin, monoacetylated myricetin and diacetylated myricetin respectively. Figure S2: HPLC chromatogram of the enzymatic acetylation of quercetin by TLL-ZnOFe monitored at 370 nm. The peaks at 11.21, 12.88 and 15.07 min correspond to quercetin, monoacetylated quercetin and diacetylated quercetin respectively. The peak at 17.02 min could be a trace of triacetylated product. Figure S3: HPLC chromatogram of the enzymatic acetylation of luteolin by TLL-ZnOFe monitored at 348 nm. The peaks at 11.02, 12.62 and 14.77 min correspond to luteolin, monoacetylated luteolin and diacetylated luteolin respectively. Figure S4: HPLC chromatogram of the enzymatic acetylation of naringenin by TLL-ZnOFe monitored at 288 nm. The peaks at 12.53 and 15.05 min correspond to naringenin and monoacetylated naringenin respectively. Figure S5: HPLC chromatogram of the enzymatic acetylation of fisetin by TLL-ZnOFe monitored at 360 nm. The peak at 9.58, 11.33, and 13.59 min correspond to fisetin, monoacetylated fisetin and diacetylated fisetin respectively. Figure S6: HPLC chromatogram of the enzymatic acetylation of morin by TLL-ZnOFe monitored at 254 nm. The peaks at 10.62, 13.13 and 15.25 min correspond to morin, morin monoacetylated and morin diacetylated respectively. Figure S7: Mass spectra of flavonoids and their respective products acquired in positive source of atmospheric pressure chemical ionization (APCI). Figure S8. Superposition of a selected region of the 1H-NMR spectra of luteolin (red) and the mixture of the acylation of luteolin by TLL-ZnOFe after 72 h (blue). Figure S9. Selected region of the 1H-NMR spectra of the mixture of the acylation of luteolin by TLL-ZnOFe after 72 h. Figure S10. Superposition of a selected region of the 1H-NMR spectra of morin (red) and the mixture of the acylation of morin by TLL-ZnOFe after 72 h (blue). Figure S11. Selected region of the 1H-NMR spectra of the mixture of the acylation of morin by TLL-ZnOFe after 72 h. Figure S12. Superposition of a selected region of the 1H-NMR spectra of fisetin (red) and the mixture of the acylation of fisetin by TLL-ZnOFe after 72 h (blue). Figure S13. Selected region of the 1H-NMR spectra of the mixture of the acylation of fisetin by TLL-ZnOFe after 72 h.
