# Supplementary material for: A Study on the Regioselective Acetylation of Flavonoid Aglycons Catalyzed by Immobilized Lipases
Source: Biomolecules. 2024 Jul 24;14(8):897. doi: 10.3390/biom14080897 (PMC11352720; doi:10.3390/biom14080897)
Supplement: Supplementary file 1 [file biomolecules-14-00897-s001.zip › biomolecules-3086970-supplementary.pdf]

biomolecules

MDPI

Supplementary Materials

A Study on the Regioselective Acetylation of Flavonoid Aglycons Catalyzed by Immobilized Lipases

Angelos Papanikolaou [1], Alexandra V. Chatzikonstantinou [1,*], Renia Fotiadou [1], Aliki Tsakni [2], Dimitra Houhoula [2], Angeliki C. Polydera [1], Ioannis V. Pavlidis [3] and Haralambos Stamatis [1,*]

[1] Laboratory of Biotechnology, Department of Biological Applications and Technology, University of Ioannina, 45110 Ioannina, Greece; aggelos_pap93@outlook.com (A.P.); renia.fotiadou@gmail.com (R.F.); apolyder@uoi.gr (A.C.P.)
[2] Department of Food Science and Technology, University of West Attica, 12243 Athens, Greece; aliki_tsak@yahoo.gr (A.T.); dhouhoula@uniwa.gr (D.H.)
[3] Department of Chemistry, University of Crete, Voutes University Campus, 70013 Heraklion, Greece; ipavlidis@uoc.gr
* Correspondence: alexandra_xatzi@hotmail.com (A.V.C.); hstamati@uoi.gr (H.S.)

Table S1: Effect of enzyme to nanosupport mass ratio on the immobilization yield and the activity of the immobilized nanobiocatalysts.

Enzyme:Nanosupport mass ratio	CaLB-ZnOFe		TLL-ZnOFe	
	Immobilization yield (%)	Activity (U/mg nanobiocatalyst)	Immobilization yield (%)	Activity (U/mg nanobiocatalyst)
1:4	59.4 ± 2.7	0.9 ± 0.3	63.7 ± 3.3	1.2 ± 0.5
1:2	44.3 ± 3.2	1.4 ± 0.4	48 ± 2.8	1.3 ± 0.3
1:1	32.9 ± 1.8	2.6 ± 0.6	37 ± 2.5	2.7 ± 0.4
2.5:1	23.6 ± 2.2	4.3 ± 1	26.6 ± 3.5	4.7 ± 0.9
5:1	16.3 ± 1.2	5.8 ± 0.6	18.1 ± 1.8	6.7 ± 0.7

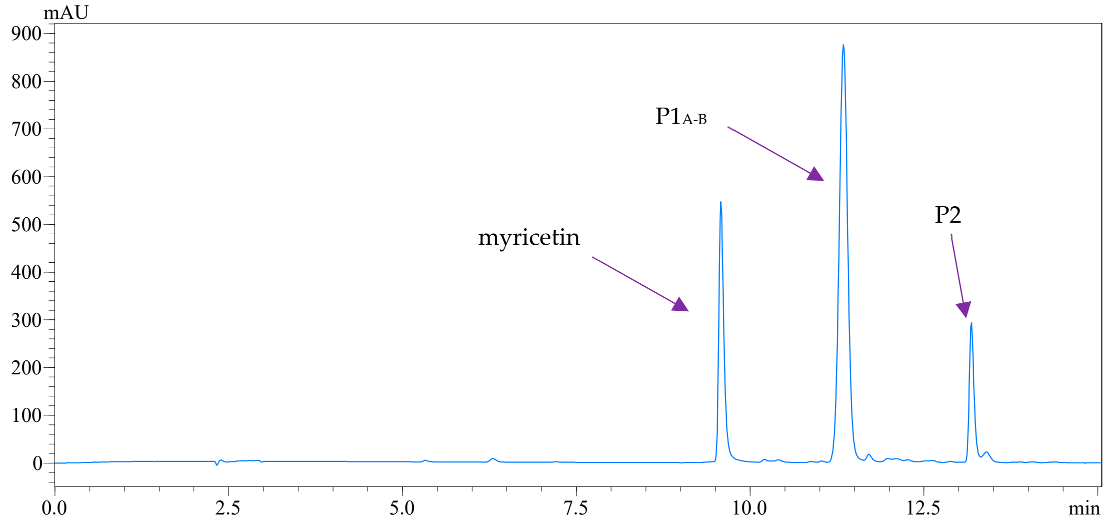

Figure S1: HPLC chromatogram of the enzymatic acetylation of myricetin by TLL-ZnOFe monitored at 370 nm. The peaks at 9.57, 11.33 and 13.19 min correspond to myricetin, monoacetylated myricetin and diacetylated myricetin respectively.

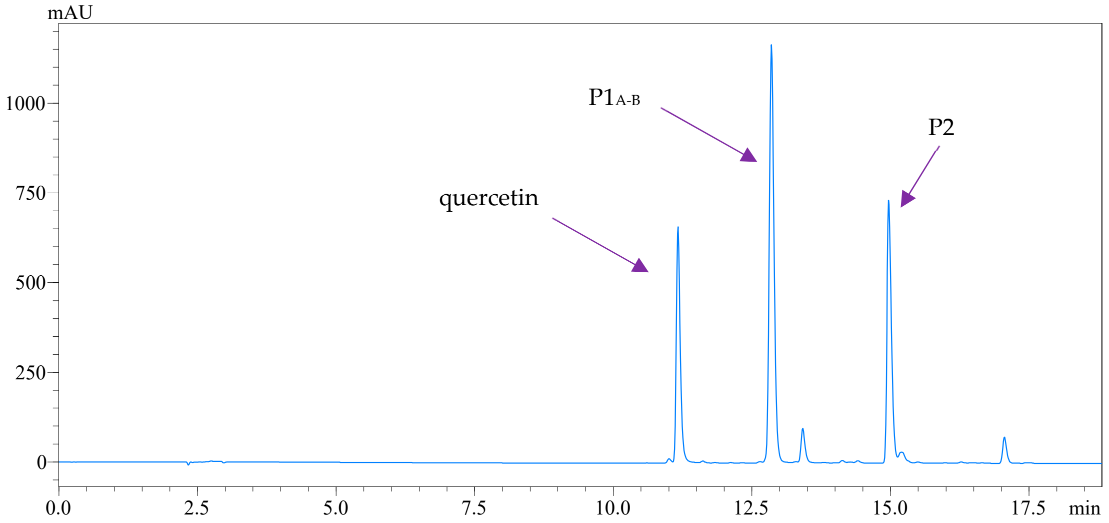

Figure S2: HPLC chromatogram of the enzymatic acetylation of quercetin by TLL-ZnOFe monitored at 370 nm. The peaks at 11.21, 12.88 and 15.07 min correspond to quercetin, monoacetylated quercetin and diacetylated quercetin respectively. The peak at 17.02 mins could be a trace of triacetylated product.

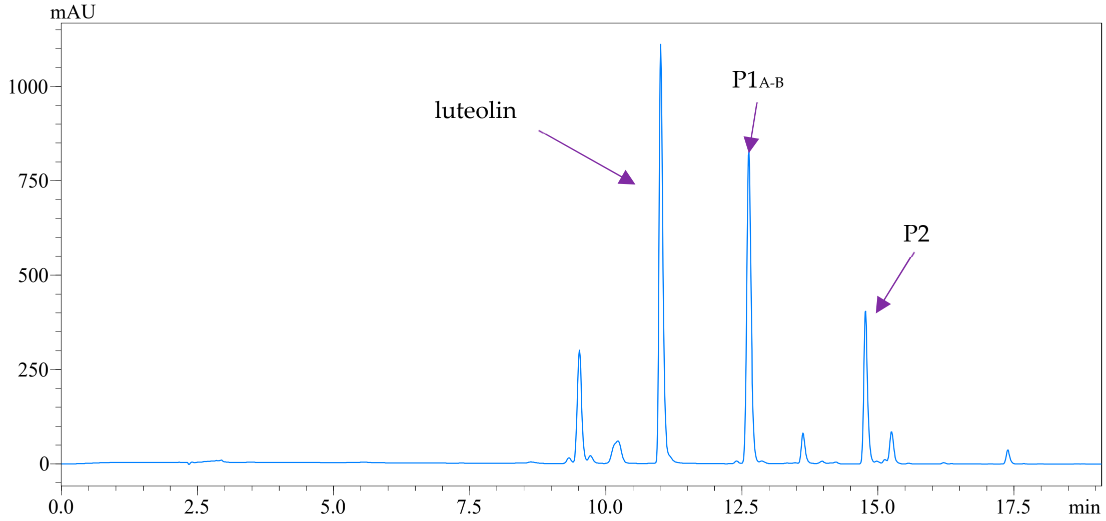

Figure S3: HPLC chromatogram of the enzymatic acetylation of luteolin by TLL-ZnOFe monitored at 348 nm. The peaks at 11.02, 12.62 and 14.77 min correspond to luteolin, monoacetylated luteolin and diacetylated luteolin respectively.

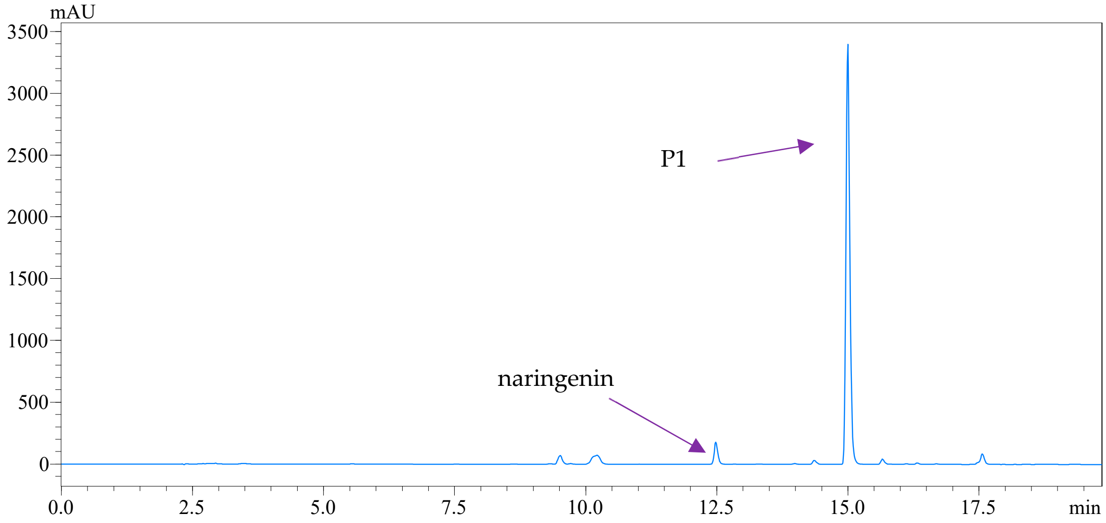

Figure S4: HPLC chromatogram of the enzymatic acetylation of naringenin by TLL-ZnOFe monitored at 288 nm. The peaks at 12.53 and 15.05 min correspond to naringenin and monoacetylated naringenin respectively.

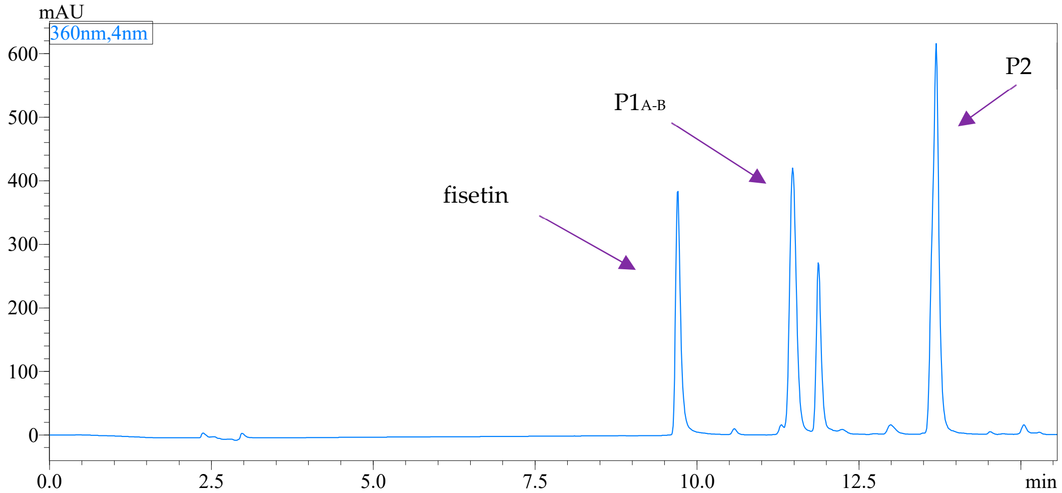

Figure S5: HPLC chromatogram of the enzymatic acetylation of fisetin by TLL-ZnOFe monitored at 360 nm. The peak at 9.58, 11.33, and 13.59 min correspond to fisetin, monoacetylated fisetin and diacetylated fisetin respectively.

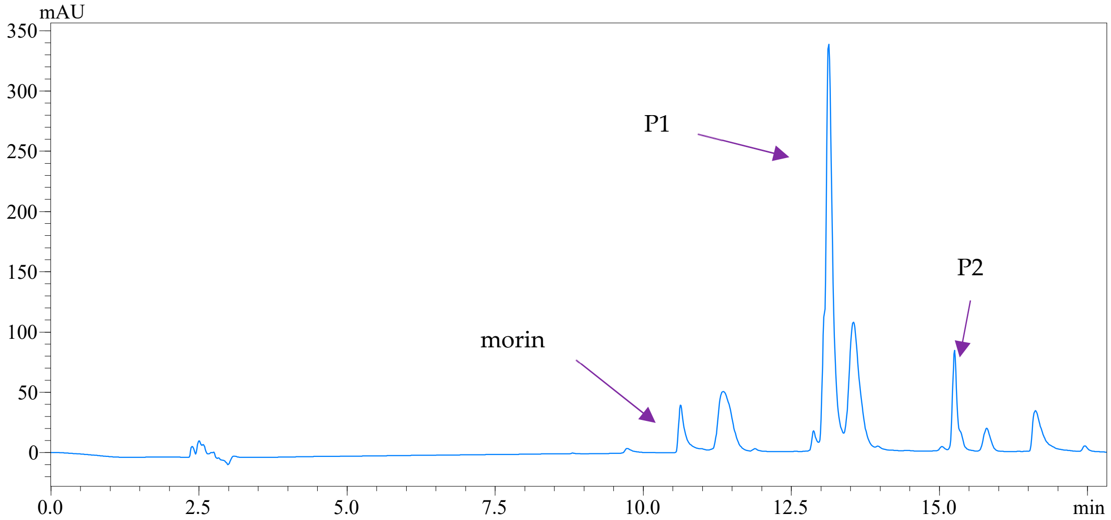

Figure S6: HPLC chromatogram of the enzymatic acetylation of morin by TLL-ZnOFe monitored at 254 nm. The peaks at 10.62, 13.13 and 15.25 min correspond to morin, morin monoacetylated and morin diacetylated respectively.

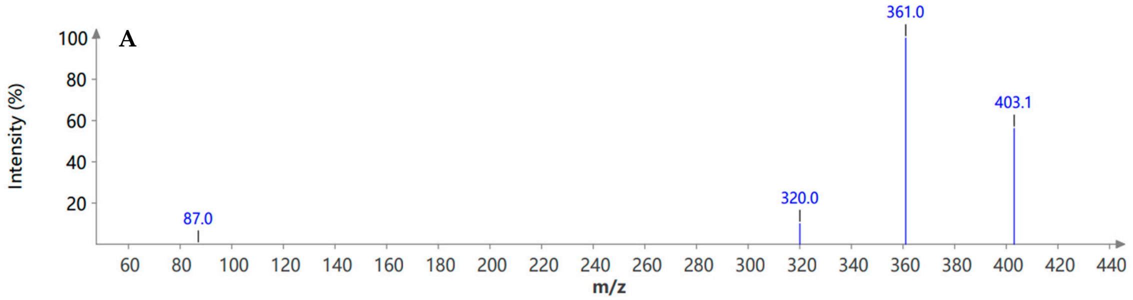

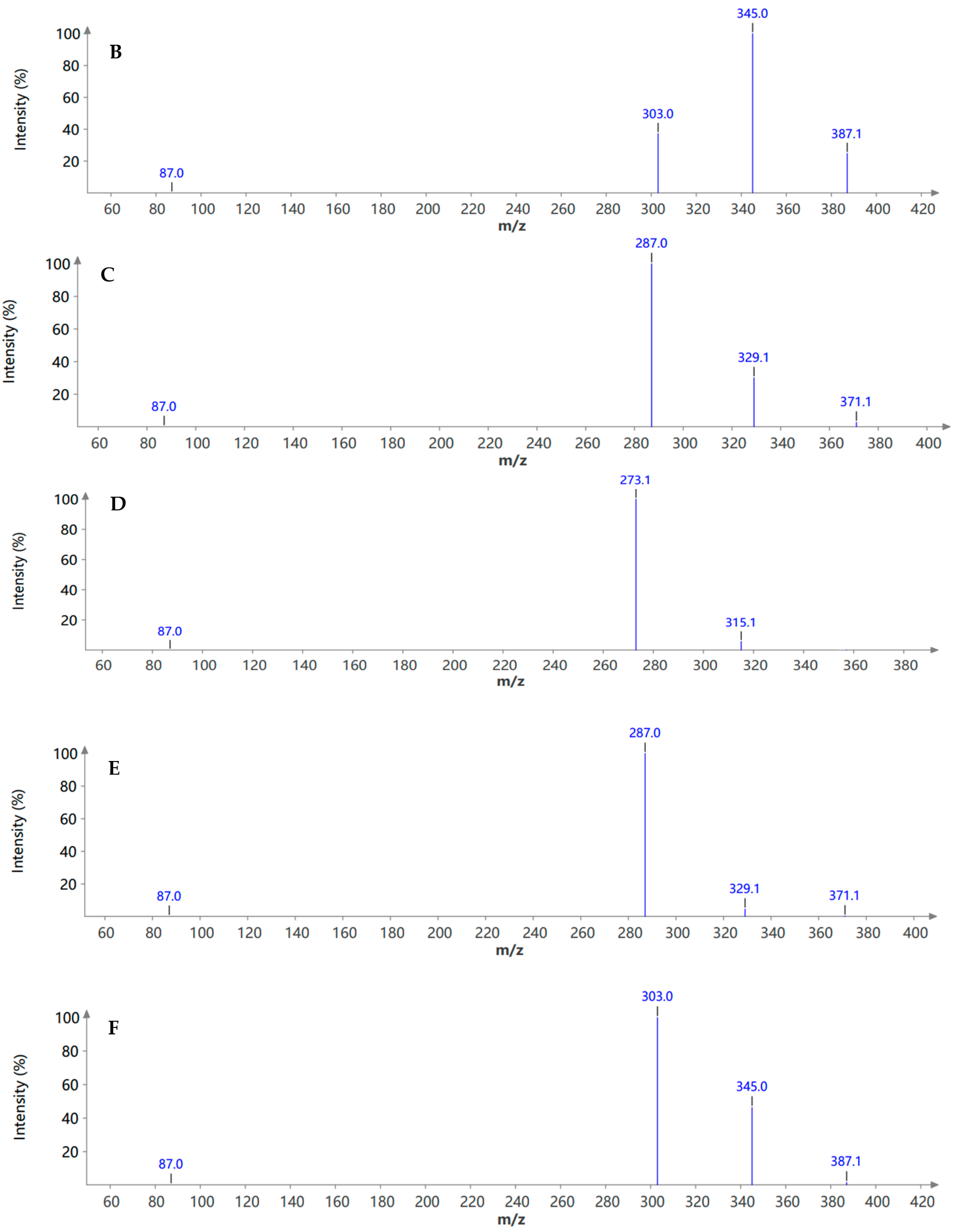

Figure S7: Mass spectra of flavonoids and their respective products acquired in positive source of atmospheric pressure chemical ionization (APCI).

A: Myricetin, B: Quercetin, C: Luteolin, D: Naringenin, E: Fisetin, F: Morin

Table S2: Flavonoids and their derivatives with their expected masses (m/z)

**Compound**	**m/z**$^{\alpha}$
Myricetin	320
mono-acetylated	361
di-acetylated	403.1
Quercetin	303
mono-acetylated	345
di-acetylated	387
Luteolin	287
mono-acetylated	329
di-acetylated	371
Naringenin	273.1
mono-acetylated	315.1
Fisetin	287
mono-acetylated	329
di-acetylated	371
Morin	303
mono-acetylated	345
di-acetylated	387

$^{\alpha}$[M+H]

^{1}H and ^{13}C NMR chemical shifts (δ, ppm) of the following compounds:

Chemical shifts of ^{1}H and ^{13}C NMR (δ, ppm) of myricetin: ^{1}H-NMR (500 MHz DMSO-d_6, 25 °C): δ = 6.21 (d, $^{4}J_{H,H}$=1.84Hz, 1H, 6c-H), 6.40 (d, $^{4}J_{H,H}$=1.84Hz, 1H, 8c-H), 7.28 (s, 2H, 2' c-H, 6'c-H), 8.84(s, 2H, 4'c-OH, 5'c-OH), 9.25(s, 2H, 3'c-OH, 5'c-OH), 9.37(s, 1H, 3c-OH), 10.81 (s, 1H, 7c-OH), 12.53 (s, 1H, 5c-OH)ppm; ^{13}C-NMR: δ = 93.86 (C8), 98.79 (C6), 103.83(C10), 107.92(C2', C6'), 121.58(1'), 136.69(C3), 139.00(C4'), 146.52(C3',C5'), 147.68(C2), 156.96(C9), 161.60(C5), 164.61(C7), 176.61(C4)ppm;

Chemical shifts of ^{1}H and ^{13}C NMR (δ, ppm) of 4'-myricetin acetate: ^{1}H-NMR, (500 MHz DMSO-d_6, 25 °C): δ = 2.28 (s, 3H 8'c-H), 6.24 (d, $^{4}J_{H,H}$=1.40Hz, 1H, 6c-H), 6.41 (d, $^{4}J_{H,H}$=1.40Hz, 1H, 8c-H), 7.28 (s, 2H, 2'c-H, 6'c-H), 9.22(s, 1H, 5'c-OH), 9.66(s, 1H, 3c-OH), 9.82(s, 1H, 3'c-OH), 10.90 (s, 1H, 7c-OH), 12.41 (s, 1H, 5c-OH)ppm; ^{13}C-NMR: δ = 21.23(C8'), 93.96(C8), 99.01(C6), 104.06(C10), 107.64(C2', C6'), 121.70(C1'), 128.98(C4'), 137.80(C3), 146.36(C2), 150.94(C3',C5'), 157.03(C9), 161.63(C5), 165.07(C7), 168.98 (7'), 177.04(C4)ppm;

Chemical shifts of ^{1}H and ^{13}C NMR (δ, ppm) of 3'-myricetin acetate: ^{1}H NMR (500 MHz DMSO-d_6, 25 °C): δ = 2.32 (s, 3H 8'c-H), 6.23 (d, $^{4}J_{H,H}$=1.41Hz, 1H, 6c-H), 6.45 (d, $^{4}J_{H,H}$=1.41Hz, 1H, 8c-H), 7.42 (s, 1H, 2'c-H), 7.66 (s, 1H, 6'c-H), 9.58(s, 1H, 3c-OH), 9.60(s, 1H, 4'c-OH), 9.87(s, 1H, 5'c-OH), 10.85 (s, 1H, 7c-OH), 12.46 (s, 1H, 5c-OH)ppm; ^{13}C-NMR, δ = 21.23(C8'), 94.04(C8), 99.01(C6), 103.95(C10), 113,01(C6'), 114,40(C2'), 121.70(C1'), 137.18(C3), 139.60(C4'), 140.74(C3'), 146.53(C2), 146.98(5'), 157.03(C9), 161.58(C5), 165.02(C7), 169.74(7'), 176.72(C4)ppm;

Chemical shifts of ^{1}H and ^{13}C NMR (δ, ppm) of 4′,3′-myricetin diacetate: ^{1}H NMR (500 MHz DMSO-d$_6$, 25 °C): δ = 2.28 (s, 3H, 8′c-H), 2.28 (s, 3H, 10′c-H), 6.25 (d, $^{4}J_{H,H}$=1.93, 1H, 6c-H), 6.47 (d, $^{4}J_{H,H}$=1.99, 1H, 8c-H), 7.50 (d, $^{4}J_{H,H}$=1.97, 1H, 2′c-H), 7.75 (d, $^{4}J_{H,H}$=1.92, 1H, 6′c-H), 9.90, (s, 1H, 3c-OH), 10.40 (s, 1H, 5′c-OH), 10.94 (s, 1H, 7c-OH), 12.34 (s, 1H, 5c-OH)ppm; ^{13}C NRM, δ = 21.40 (C8′), 21.40 (C10′), 94.0(C8), 99.00(C6), 104.13(C10), 113.26(C2′), 114.0(C6′), 114.3(C1′), 132.80(C3′), 132.80(C4′), 138.30(C3), 145.0(C2), 151.12(C5′), 157.12(C9), 161.61(C5), 165.0(C7), 169.0(C7′), 169.0(C9′), 177.0(C4)ppm.

Chemical shifts of ^{1}H and ^{13}C NMR (δ, ppm) of quercetin: ^{1}H NMR (500 MHz DMSO-d$_6$, 25 °C): δ = 6.22 (d, $^{4}J_{H,H}$=2.08Hz, 1H, 6c-H), 6.44 (d, $^{4}J_{H,H}$=2.08Hz, 1H, 8c-H), 6.92 (d, $^{3}J_{H,H}$=8.50Hz, 1H, 5′c-H), 7.57 (dd, $^{4}J_{H,H}$=2.23Hz, ^{3}JH,H=6.28Hz, 1H, 6′ c-H), 7.71(d, $^{4}J_{H,H}$=2.20Hz, 1H, 2′c-H), 9.37(s, 1H, 3′c-OH), 9.44(s, 1H, 3c-OH), 9.66 (s, 1H, 4′c-OH), 10.85 (s, 1H, 7c-OH), 12.54 (s, 1H, 5c-OH)ppm; ^{13}C-NMR: δ = 94.00 (C8), 98.81(C6), 103.93(C10), 105.72(C2′), 106.22(C5′), 120.62(C6′), 123.12(C1′), 136.83(C3), 146.25(C3′), 147.84(C2), 148.73(C4′), 157.44(C9), 161.90(C5), 165.00(C7), 176.93(C4)ppm;

Chemical shifts of ^{1}H and ^{13}C NMR (δ, ppm) of 3′-quercetin acetate: ^{1}H NMR (500 MHz DMSO-d$_6$, 25 °C):δ = 2.33 (s, 3H 8′c-H), 6.23(d, $^{4}J_{H,H}$=2.08Hz, 1H, 6c-H), 6.50 (d, $^{4}J_{H,H}$=2.08Hz, 1H, 8c-H), 7.12 (d, 1H, $^{3}J_{H,H}$=8.50Hz, 1H, 5′ c-H), 7.91(d, $^{4}J_{H,H}$=2.20Hz, 1H, 2′c-H), 7.99 (dd, $^{4}J_{H,H}$=2.23Hz, $^{3}J_{H,H}$=6.28Hz, 1H, 6′ c-H), 9.66(s, 1H, 3c-OH), 10.48(s, 1H, 4′c-OH), 10.89 (s, 1H, 7c-OH), 12.47 (s, 1H, 5c-OH)ppm; ^{13}C-NMR: δ = 21.4 (C8′), 94.20 (C8), 98.91(C6), 117.63(C5′), 123.22(C1′), 123.34(C2′), 127.25(6′), 137.23(C3), 139.23(C3′), 146.74(C2), 152.13(C4′), 157.23(C9), 161.94(C5), 165.20(C7), 169.90(C7′), 176.93(C4)ppm;

Chemical shifts of ^{1}H and ^{13}C NMR (δ, ppm) of 4′-quercetin acetate: ^{1}H NMR (500 MHz DMSO-d$_6$, 25 °C):δ= 2.32 (s, 3H 8′c-H), 6.25(d, $^{4}J_{H,H}$=2.08Hz, 1H, 6c-H), 6.47 (d, $^{4}J_{H,H}$=2.08Hz, 1H, 8c-H), 7.22 (d, $^{3}J_{H,H}$=8.50Hz, 1H, 5′ c-H), 7.64(dd, $^{4}J_{H,H}$=2.23Hz, $^{3}J_{H,H}$=6.28Hz, 1H, 6′c-H), 7.83 (d, $^{4}J_{H,H}$=2.20Hz, 1H, 2′ c-H), 9.78(s, 1H, 3c-OH), 10.09(s, 1H, 3′c-OH), 10.94 (s, 1H, 7c-OH), 12.41 (s, 1H, 5c-OH)ppm; ^{13}C-NMR: δ = 21.3 (C8′), 94.10 (C8), 98.91(C6), 104.20(C10), 116.72(C2′), 119.42(C6′), 124.00(5′), 130.30(C1′), 138.23(C3), 140.64(C4′), 146.43(C2), 150.00(C3′), 157.42(C9), 161.90(C5), 165.40(C7), 169.8 (c7′), 176.93(C4)ppm;

Chemical shifts of ^{1}H and ^{13}C NMR (δ, ppm) of 3′,4′-quercetin diacetate: ^{1}H NMR (500 MHz DMSO-d$_6$, 25 °C):δ = 2.33 (s, 3H 8′c-H), 2.33 (s, 3H, 10′c-H), 6.26(d, $^{4}J_{H,H}$ = 2Hz, 1H, 6c-H), 6.52 (d, $^{4}J_{H,H}$ = 2Hz, 1H, 8c-H), 7.51(d, $^{3}J_{H,H}$=8.8 Hz, 1H, 5′c-H), 8.1(d, $^{4}J_{H,H}$ = 2.1Hz, 1H, 2′c-H), 8.2(dd, $^{4}J_{H,H}$=2.1Hz, $^{3}J_{H,H}$=8.25Hz, 1H, 6′c-H), 9.97(s, 1H, 3c-OH), 10.95(s,1H, 7c-H), 12.34 (s, 1H, 5c-OH)ppm; ^{13}C: δ = 21.21 (C8′), 21.21 (C10′), 94.32 (C8), 99.07 (C6), 108.37 (C10), 123.37 (C2′), 124.68 (C5′), 126.61(C6′), 126.78(C1′), 138.2(C3), 142.85(C3′), 143.87(C4′), 144.86(C2), 153.23(C9), 161.62(C5), 165.27(C7), 169.55(C7′), 169.55(C9′), 177.15 (C4) ppm.

Chemical shifts of ^{1}H and ^{13}C NMR (δ, ppm) of naringenin: ^{1}H NMR (500 MHz DMSO-d$_6$, 25 °C):δ = 2.68 (dd, $^{4}J_{H,H}$=2.78Hz, $^{3}J_{H,H}$=17.17Hz, 1H, 3c-H), 5.42 (dd, $^{4}J_{H,H}$=12.85Hz, $^{3}J_{H,H}$=17.71Hz, 1H, 2 c-H), 5.88 (s, 2H, 6c-H, 8c-H), 6.80 (d, $^{3}J_{H,H}$=8.34Hz, 2H, 3′c-H, 5′c-H), 7.32 (d, $^{3}J_{H,H}$=8.35Hz, 2H, 2′c-H, 6′c-H), 9.59 (s, 1H, 4′ c-OH), 10.80(s, 1H, 7c-OH), 12.15(s, 1H, 5c-OH)ppm; ^{13}C-NMR: δ = 42.48 (C3), 78.91(C2), 95.93(C8), 96.62(C6), 102.60(C10), 115.92(C3′, C5′), 129.12(C2′, C6′), 158.63(C4′), 163.25(C9), 164.24(C5), 167.33(C7), 196.94(C4)ppm;

Chemical shifts of ^{1}H and ^{13}C NMR (δ, ppm) of 4′-naringenin acetate: ^{1}H NMR (500 MHz DMSO-d$_6$, 25 °C): δ = 2.28 (s, 3H, c8′-H), 2.83 (dd, $^{4}J_{H,H}$=3.12Hz, $^{3}J_{H,H}$=17.17Hz, 1H, 3c-H), 5.62 (dd, $^{4}J_{H,H}$=3.08Hz, $^{3}J_{H,H}$=12.78Hz, 1H, 2 c-H), 5.90 (s, 1H, 6c-H), 5.93 (dd, $^{4}J_{H,H}$=1.15Hz, $^{3}J_{H,H}$=13.31Hz, 2H, 6c-H, 8c-H), 7.31 (d, $^{3}J_{H,H}$=8.35Hz, 2H, 3′c-H, 5′c-H), 7.56 (d, $^{3}J_{H,H}$=8.62Hz, 2H, 2′ c-H, 6′c-H), 12.12(s, 1H,7c-OH), 12.84(s, 1H, 5c-OH)ppm; ^{13}C-NMR: δ = 21.43(C8′), 42.63 (C3), 78.21(C2), 95.93(C8), 96.62(C6), 102.60(C10), 122.32(C3′, C5′), 128.42(C2′, C6′), 136.6(C1′), 150.93(C4′), 163.45(C9), 164.24(C5), 167.33(C7), 169.9 (C7′), 196.94(C4)ppm;

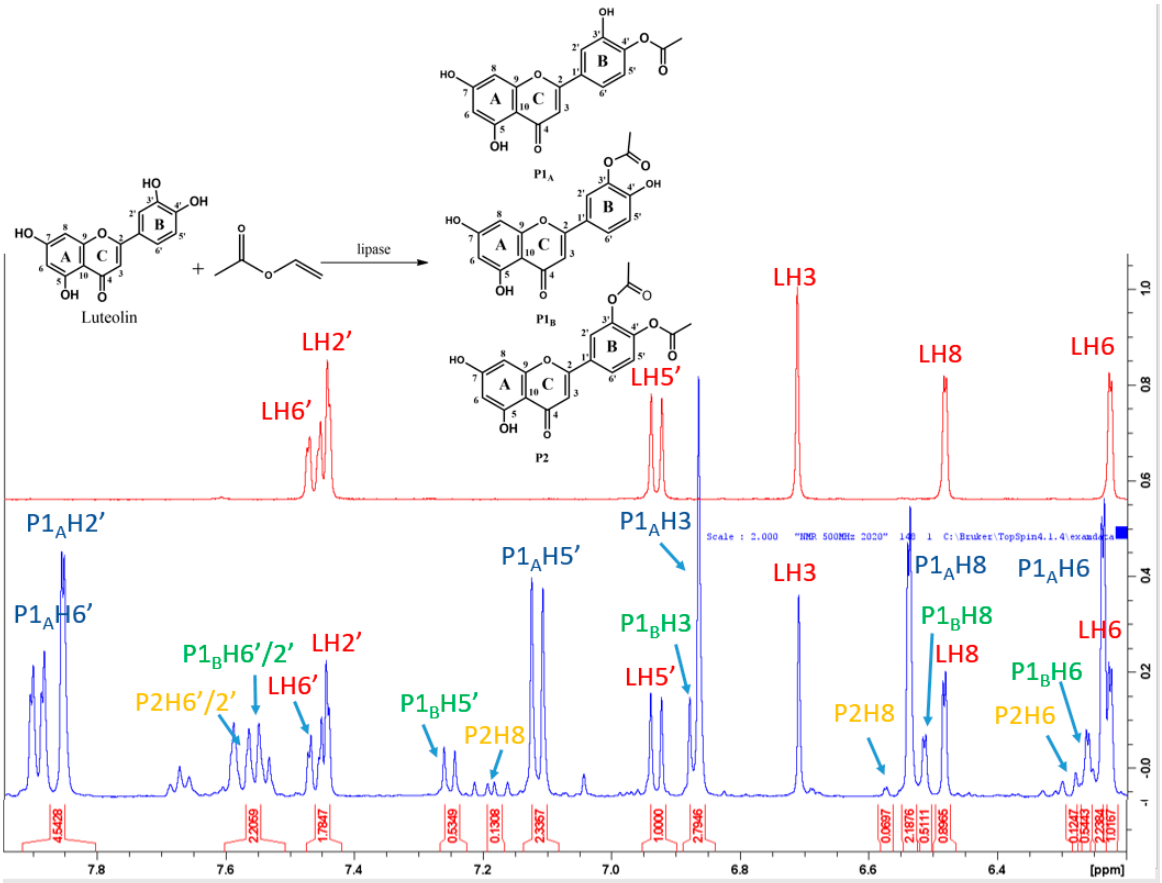

Figure S8. Superposition of a selected region of the 1H-NMR spectra of luteolin (red) and the mixture of the acylation of luteolin by TLL-ZnOFe after 72 h (blue).

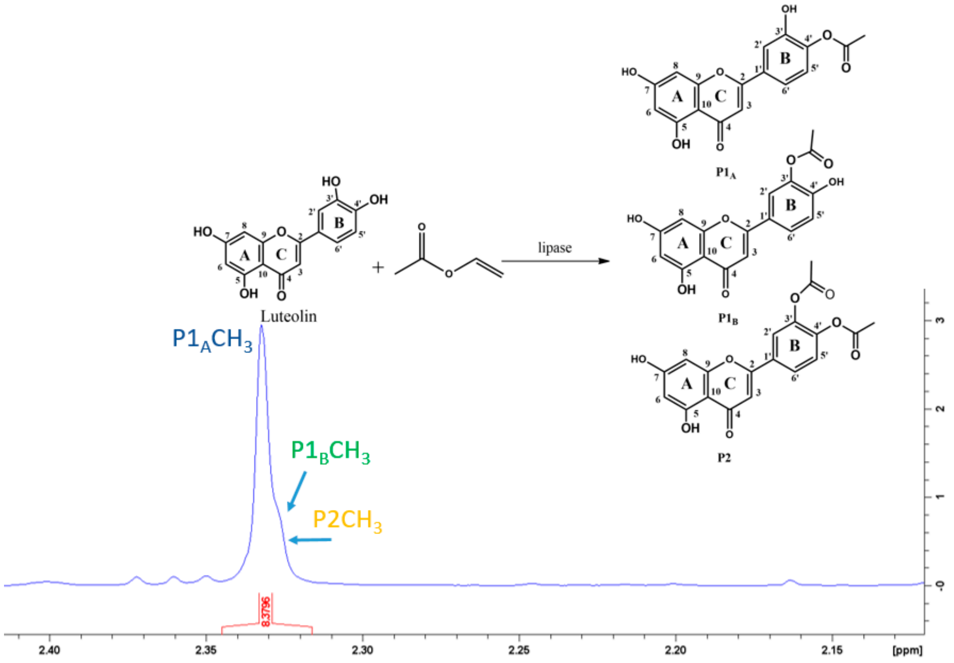

Figure S9. Selected region of the 1H-NMR spectra of the mixture of the acylation of luteolin by TLL-ZnOFe after 72h.

Chemical shifts of ^{1}H and ^{13}C NMR (δ, ppm) of luteolin: ^{1}H NMR (500 MHz DMSO-d$_6$, 25 ^{o}C):δ = 6.23 (d, $^{4}J_{H,H}$=1.80Hz, 1H, 6c-H), 6.48 (d, $^{4}J_{H,H}$=1.87Hz, 1H, 8c-H), 6.7 (s, 1H, 3c-H), 6.93 (d, $^{3}J_{H,H}$=8.31Hz, 1H, 5'c-H), 7.43 (d, $^{4}J_{H,H}$=2Hz, 1H, 2'c-H), 7.45 (d, $^{3}J_{H,H}$=8.5Hz, $^{4}J_{H,H}$=2Hz, 1H, 6'c-H), 9.44 (s, 1H, 3'c-OH), 9.95 (s, 1H, 4'c-OH), 10.87 (s, 1H, 7c-OH), 13 (s,

1H, 5c-OH)ppm; ^{13}C-NMR: δ = 94.47(C8), 99.40(C6), 103.46(C3), 104.61(C10), 114(C2'), 116.55(C5'), 119.60(C6'), 122.40(C1'), 146.50(C3'), 150.60(C4'), 158.20(C9), 162.34(C5), 164.80(C2), 165.05(C7), 182.50(C4)ppm.

Chemical shifts of ^{1}H and ^{13}C NMR (δ, ppm) of 4'-luteolin acetate: ^{1}H NMR (500 MHz DMSO-d$_6$, 25 °C):δ = 2.33 (s, 1H, 8'c-H), 6.23 (d, $^{4}J_{H,H}$=1.93Hz, 1H, 6c-H), 6.53 (d, $^{4}J_{H,H}$=1.83Hz, 1H, 8c-H), 6.86 (s, 1H, 3c-H), 7.11 (d, $^{3}J_{H,H}$=8.7Hz, 1H, 5'c-H), 7.84 (d, $^{4}J_{H,H}$=1.88Hz, 1H, 2'c-H), 7.88 (d, $^{3}J_{H,H}$=8.5Hz, $^{4}J_{H,H}$=2Hz, 1H, 6'c-H), 10.90 (s, 1H, 7c-OH), 10.20 (s, 1H, 3'c-OH), 12.95 (s, 1H, 5c-OH)ppm; ^{13}C-NMR: δ = 21.30(C8'), 94.57(C8), 99.57(C6), 104(C3), 104.64(C9), 117.8(C5'), 122.3(C2'), 122.43(C1'), 126.14(C6'), 139.57(C3'), 153.8(C4'), 158.16(C10), 162.22(C5), 163.44(C2), 165(C7), 169.52(C7'), 182.70(C4)ppm.

Chemical shifts of ^{1}H and ^{13}C NMR (δ, ppm) of 3'-luteolin acetate: ^{1}H NMR (500 MHz DMSO-d$_6$, 25 °C):δ = 2.33 (s, 1H, 8'c-H), 6.26 (d, $^{4}J_{H,H}$=1.95Hz, 1H, 6c-H), 6.51 (d, $^{4}J_{H,H}$=1.76Hz, 1H, 8c-H), 6.87 (s, 1H, 3c-H), 7.24 (d, 1H, $^{3}J_{H,H}$=8.16Hz, 5'c-H), 7.54 (d, $^{4}J_{H,H}$=1.87Hz, 1H, 2'c-H), 7.55 (d, $^{3}J_{H,H}$=8.5Hz, $^{4}J_{H,H}$=2,02, 1H, 6'c-H), 10.68 (s, 1H, 4'c-OH), 10.96 (s, 1H, 7c-OH), 12.86 (s, 1H, 5c-OH)ppm; ^{13}C-NMR: δ = 21.35(C8'), 94.55(C8), 99.66(C6), 104.64(C9), 105.59(C3), 122.3(C1'), 122.4(C2'), 124.53(C5'), 126.22(C6'), 139.40(C4'), 153.6(C3'), 158.16(C10), 162.21(C5), 163.44(C2), 165(C7), 169.5(C7'), 182.70(C4)ppm.

Chemical shifts of ^{1}H and ^{13}C NMR (δ, ppm) of 3',4'-luteolin diacetate: ^{1}H NMR (500 MHz DMSO-d$_6$, 25 °C):δ = 2.31 (s, 1H, 8'c-H), 2.31 (s, 1H, 10'c-H), 6.28 (d, $^{4}J_{H,H}$=2.3, 1H, 6c-H), 6.57 (d, $^{4}J_{H,H}$=2.1, 1H, 8c-H), 6.88 (s, 1H, 3c-H), 7.18 (d, $^{3}J_{H,H}$=8.5Hz, 1H, 5'c-H), 7.54 (d, $^{4}J_{H,H}$=2Hz, 1H, 2'c-H), 7.55 (d, $^{3}J_{H,H}$=8.3Hz, $^{4}J_{H,H}$=2.2Hz, 1H, 6'c-H), 10.98 (s, 1H, 7c-OH), 12.8 (s, 1H, 5c-OH)ppm; ^{13}C-NMR: δ = 21.35(C8'), 21.35(C10'), 94.6(C8), 99.68(C6), 104.85(C9), 105.6(C3), 122.4(C1'), 122.45 (C2'), 124.6(C5'), 126.3 (C6'), 143.5 (C3'), 144.6(C4'), 158.2(C10), 162.24(C5), 163.47(C2), 165.5(C7), 169.8(C7'), 169.8 (C9') 182.9(C4)ppm.

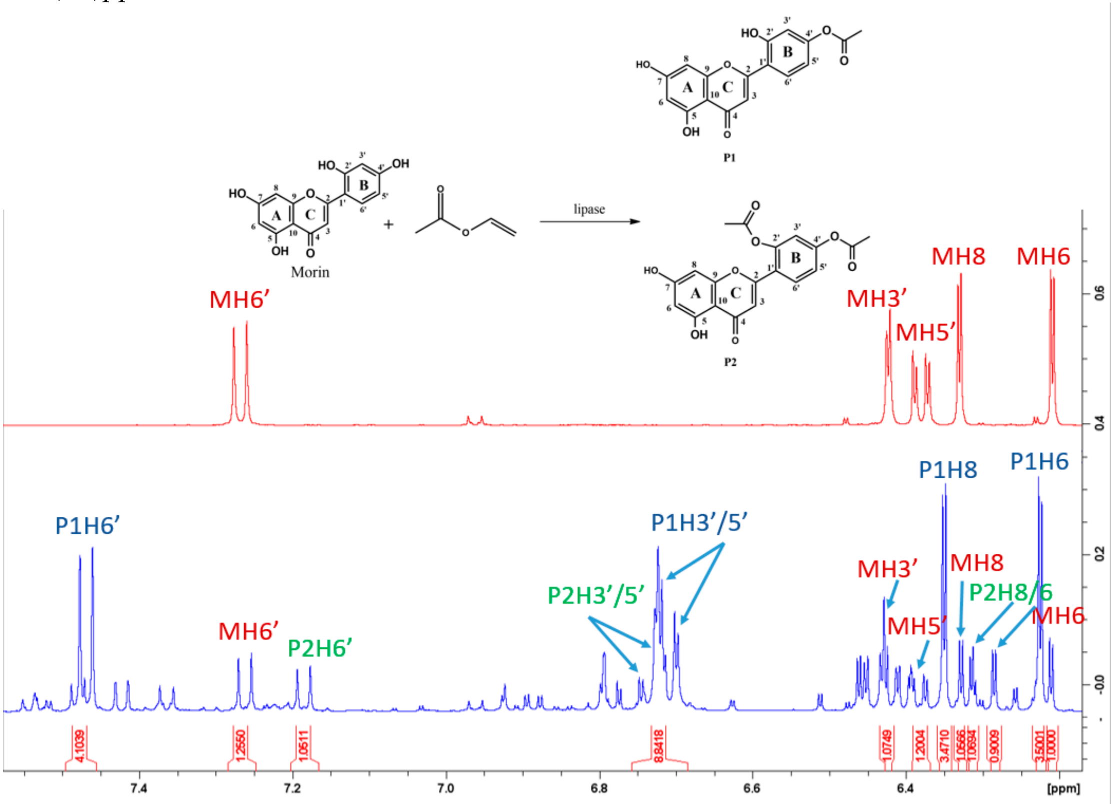

Figure S10. Superposition of a selected region of the ^{1}H-NMR spectra of morin (red) and the mixture of the acylation of morin by TLL-ZnOFe after 72h (blue).

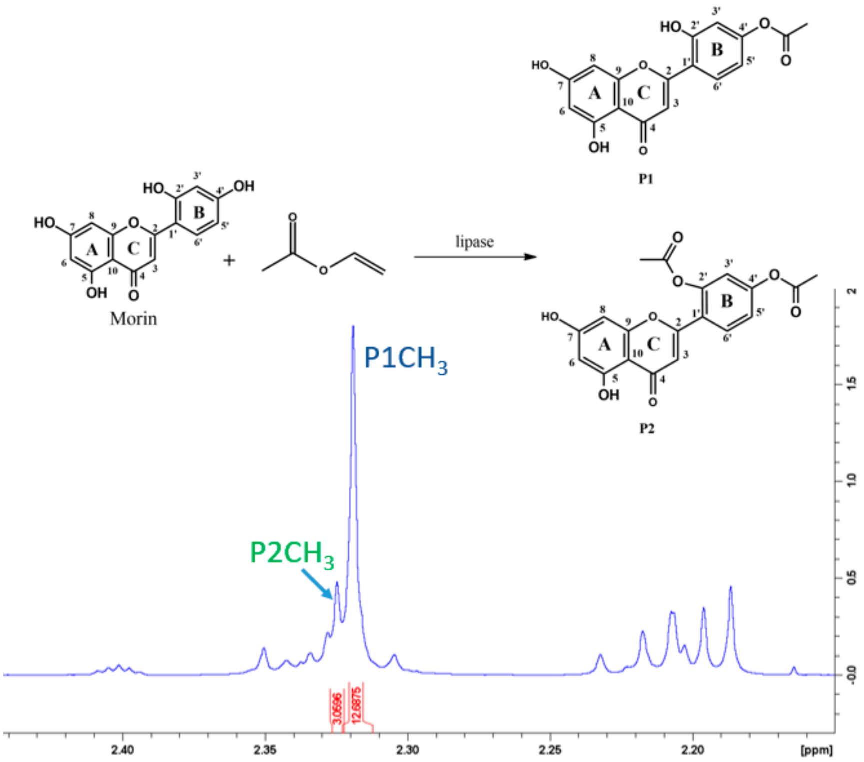

Figure S11. Selected region of the ^{1}H-NMR spectra of the mixture of the acylation of morin by TLL-ZnOFe after 72 h.

Chemical shifts of ^{1}H and ^{13}C NMR (δ, ppm) of morin: ^{1}H NMR (500 MHz DMSO-d$_6$, 25 ^{o}C): δ = 6.20 (d, $^{4}J_{H,H}$=2.07, 1H, 6c-H), 6.33 (d, $^{4}J_{H,H}$=2.07, 1H, 8c-H), 6.38 (s, 1H, 5'c-H), 6.42 (d, $^{4}J_{H,H}$=2.27, 1H, 3'c-H), 7.30 (d, $^{4}J_{H,H}$=8.51, 1H, 6'c-H), 8.90 (s, 1H, 2'c-OH), 9.75 (s, 1H, 3c-OH), 9.81 (s, 1H, 4'c-OH), 12.57 (s, 1H, 7c-OH), 12.68 (s, 1H, 5c-OH)ppm; ^{13}C-NMR: δ = 93.84(C8), 98.50(C6), 103.60(C3'), 104.60(C10), 107.30(C5'), 110.24(C1'), 132(C6'), 136.54(C3), 147.70(C2), 157.50(C9), 157.9(C2'), 161.31(C4'), 161.77(C5), 164.22(C7), 177(C4)ppm.

Chemical shifts of ^{1}H and ^{13}C NMR (δ, ppm) of 4'-morin acetate: ^{1}H NMR (500 MHz DMSO-d$_6$, 25 ^{o}C): δ = 2.31 (s, 3H, 8'c-H), 6.22 (d, $^{4}J_{H,H}$=2.05Hz, 1H, 6c-H), 6.34(d, $^{4}J_{H,H}$=2.05Hz, 1H, 8c-H), 6.70 (dd, $^{4}J_{H,H}$=2.2Hz, $^{3}J_{H,H}$=8.05Hz, 1H, 5'c-H), 6.72 (d, $^{4}J_{H,H}$=2Hz, 1H, 3'c-H), 7.47 (d, $^{3}J_{H,H}$=8.25Hz, 1H, 6'c-H), 10.78 (s, 1H, 7c-OH), 12.63 (s, 1H, 5C-OH) ppm; ^{13}C-NMR: δ = 21.65(C8'), 98.18 (C8), 98.81 (C6), 104.67 (C10), 110.74 (C3'), 112.84 (C5'), 116.94 (C1'), 132.05 (C2'), 153.58 (C4'), 157.85 (C9), 157.87 (C2'), 161.86 (C5), 164.71 (C7), 169.9 (C7'), 177.44 (C4) ppm.

Chemical shifts of ^{1}H and ^{13}C NMR (δ, ppm) of 2',4'-morin diacetate: ^{1}H NMR (500 MHz DMSO-d$_6$, 25 ^{o}C): δ = 2.32 (s, 3H, 8'c-H), 2.31 (s, 3H, 10'c-H), 6.28 (d, $^{4}J_{H,H}$=2.08Hz, 1H, 6c-H), 6.31 (d, $^{4}J_{H,H}$=2.08Hz, 1H, 8c-H), 6.7 (dd, $^{4}J_{H,H}$=2.2Hz, $^{3}J_{H,H}$=8.5Hz, 1H, 5'c-H), 6.74 (d, $^{4}J_{H,H}$=2.1Hz, 1H, 3'c-H), 7.18 (d, $^{3}J_{H,H}$=8.5Hz, 1H, 6'c-H), 10.73 (s, 1H, 7c-H), 12.34 (s, 1H, 5c-OH) ppm; ^{13}C-NMR: δ = 21.65 (C10'), 21.58 (C8'), 94 (C8), 99.68 (C6), 104.76 (C10), 111.27 (C3'), 112.71 (C5'), 116.90 (C1'), 131.7 (C6'), 157.73 (C9), 158.22 (C2'), 162.14 (C5), 164.58 (C7), 169.83 (C7',C9'), 175.83 (C4) ppm.

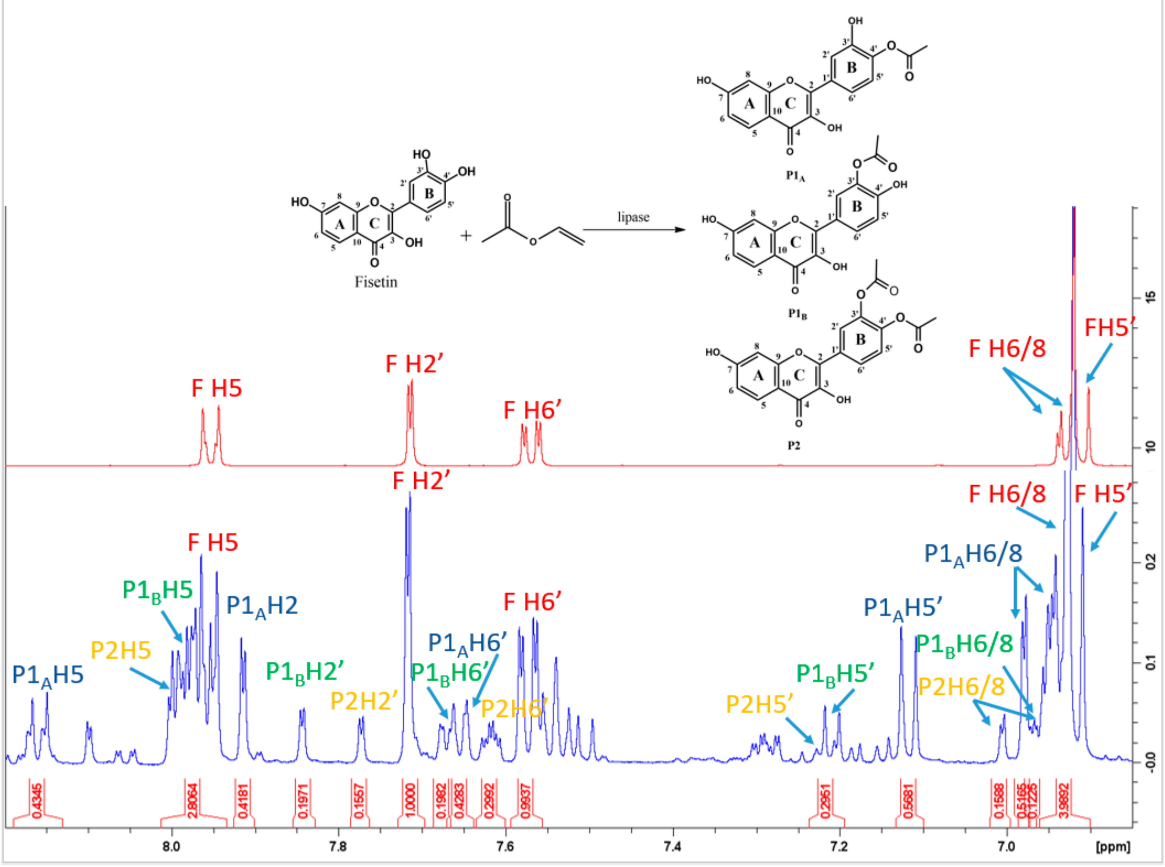

Figure S12. Superposition of a selected region of the ^{1}H-NMR spectra of fisetin (red) and the mixture of the acylation of fisetin by TLL-ZnOFe after 72h (blue).

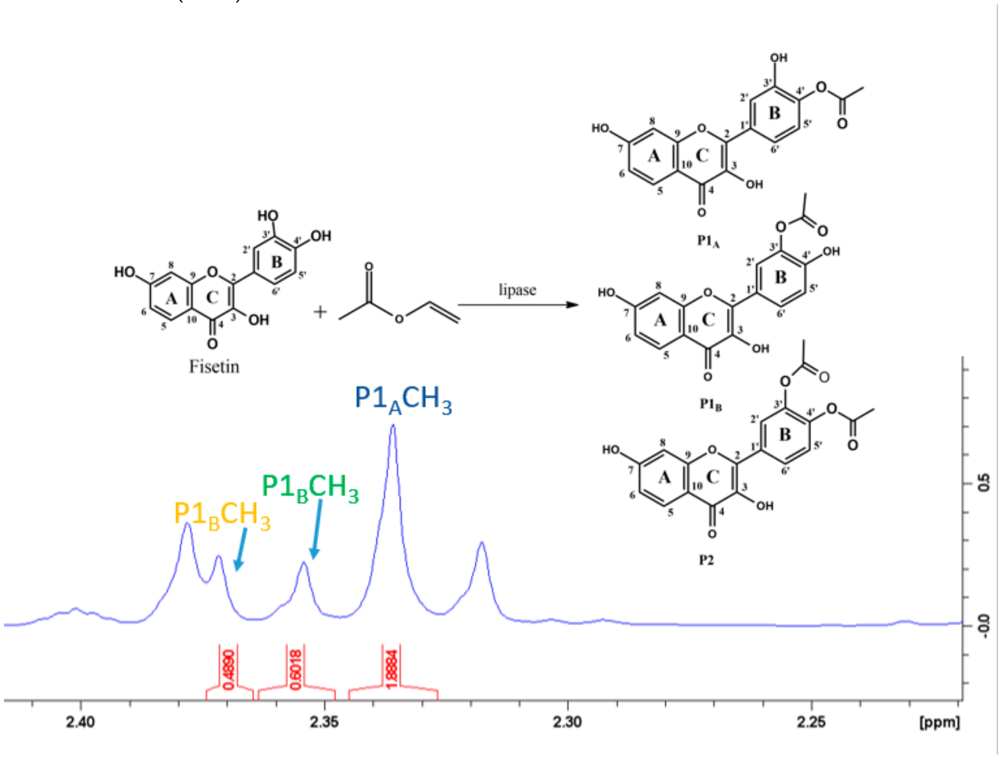

Figure S13. Selected region of the ^{1}H-NMR spectra of the mixture of the acylation of fisetin by TLL-ZnOFe after 72h.

Chemical shifts of ^{1}H and ^{13}C NMR (δ, ppm) of fisetin: ^{1}H NMR (500 MHz DMSO-d$_6$, 25 °C): δ =6.91(d, $^{3}J_{H,H}$=10.39, 1H, 5'c-H), 6.93 (dd, $^{4}J_{H,H}$ = 2.2Hz, $^{3}J_{H,H}$ =8Hz. 1H, 6c-H), 6.93(d, $^{4}J_{H,H}$=2.27Hz, 1H, 8c-H), 7.57 (dd, $^{4}J_{H,H}$=2.2Hz, $^{3}J_{H,H}$=8.54Hz,

1H, 6'c-H), 7.72 (d, $^4J_{H,H}$=2.2Hz, 1H, 2'c-H), 7.95 (d, $^3J_{H,H}$=9.39Hz, 1H, 5c-H), 9.06 (s, 1H, 3c-OH), 9.30 (s, 1H, 4'c-OH), 9.54(s, 1H, 3'c-OH), 10.78 (s, 1H, 7c-OH) ppm; ^{13}C NMR: δ = 102.4(C8), 115.29 (C10), 115.34 (C2'), 115.68 (C6), 115.85 (C5'), 120.06 (C6'), 123.2 (C1'), 126.88 (C5), 138.07 (C3), 145.92 (C2), 145.92 (C3'), 148.06 (C4'), 157.17 (C9), 163.11 (C7), 172.98 (C4) ppm.

Chemical shifts of ^{1}H and ^{13}C NMR (δ, ppm) of 4'-fisetin acetate: ^{1}H NMR (500 MHz DMSO-d_6, 25 °C: δ = 2.33 (s, 3H, 8'C-H), 6.94 (dd, $^4J_{H,H}$=2.7Hz, $^3J_{H,H}$=8Hz, 1H, 6c-H), 6.95 (d, $^4J_{H,H}$=2.27Hz, 1H, 8c-H), 7.11 (d, $^3J_{H,H}$ = 8.2Hz, 1H, 5'c-H), 7.65 (dd, $^4J_{H,H}$= 2.2Hz, $^3J_{H,H}$= 8.1Hz, 1H, 6'c-H), 7.91 (d, $^4J_{H,H}$= 2.2Hz, 1H, 2'c-H), 7.96 (d, $^3J_{H,H}$ = 8.73, 1H, 5c-H), 9.10 (s, 1H, 3c-H), 9.65 (s, 1H, 3'c-OH), 10.81 (s, 1H, 7c-OH) ppm; ^{13}C-NMR: δ = 21.19 (C8'), 102 (C8), 115.25 (C6), 119.13 (C6'), 122.96 (C2'), 123.72 (C5'), 126.9 (C5), 127.45 (C1'), 134.2 (C3), 139.5 (C4'), 144.6 (C2), 151.4 (C3'), 157.3 (C9), 163.26 (C7), 169.49 (C7'), 173.02 (C4) ppm.

Chemical shifts of ^{1}H and ^{13}C NMR (δ, ppm) of 3'-fisetin acetate: ^{1}H NMR (500 MHz DMSO-d_6, 25 °C: δ = 2.35 (s, 3H, 8'c-H), 7.00 (dd, $^4J_{H,H}$= 2.7Hz, $^3J_{H,H}$= 8Hz, 1H, 6c-H), 6.97 (s, 1H, 8c-H), 7.21 (s, 1H, 5'cH), 7.67 (dd, $^4J_{H,H}$= 2.3, $^3J_{H,H}$= 8.2, 1H, 6'c-H), 7.84 (d, $^4J_{H,H}$= 2.2Hz, 1H, 2'c-H), 7.98 (d, $^3J_{H,H}$ = 8.72, 1H, 5c-H), 9.30 (s, 1H, 3c-OH), 9.42 (s, 1H, 4'c-OH) ppm; ^{13}C-NMR: δ = 21.28 (C8'), 102.20 (C8), 115.3 (C6), 119.23 (C6'), 122.94 (C2'), 123.73 (C5'), 126.95 (C5), 127.5 (C1'), 134.2 (C3), 140.1 (C3'), 144.6 (C2), 151.53 (C4'), 157.4 (C9), 169.5 (C7'), 173.02 (C4) ppm.

Chemical shifts of ^{1}H and ^{13}C NMR (δ, ppm) of 3',4'- fisetin diacetate: ^{1}H NMR (500 MHz DMSO-d_6, 25 °C: δ = 2.37 (s, 3H, 8'c-H), 2.33 (s, 3H, 10'c-H), 6.98 (dd, $^4J_{H,H}$=2.3Hz, $^3J_{H,H}$=8.2Hz, 1H, 6 c-H), 7 (d, $^4J_{H,H}$=2.25Hz, 1H, 8 c-H), 7.22 (s, 1H, 5'c-H), 7.61 (dd, $^4J_{H,H}$=2.3Hz, $^3J_{H,H}$=8.3Hz, 1H, 6'c-H), 7.7 (d, $^4J_{H,H}$=2.3Hz, 1H, 2'c-H), 8 (d, $^3J_{H,H}$=8.7Hz, 1H, 5 c-H), 9.14 (s, 1H, 3 c-OH), 10.9 (s, 1H, 7 c-OH) ppm; ^{13}C NMR: δ = 21.16 (C8'), 21.16 (C10'), 102.53 (C8), 115 (C2'), 115.52 (C6), 120.5 (C6'), 123.75 (C5'), 126.98 (C5), 127.8 (C1'), 136.5 (C3), 145.9 (C2), 147.4 (C3'), 148.7 (C4'), 157.2 (C9), 163.3 (C7) 169.59 (C7'), 169.59 (C9'), 172.9 (C4) ppm.

Table S3: Molecular interactions between morin and acetylated morin within the active site of CaLB. Distance interactions between carbon atoms and distance between donor and acceptor atoms (hydrogen bonds) are displayed in the 4th and 5th row respectively.

Compound	Type of interaction	AA	Distance (Å)	Distance D-A (Å)	Protein donor	Side Chain
Morin	Hydrophobic interactions	Ser105	3.72			
		Leu144	3.80			
		Ile 189	3.61			
		Leu278	3.21			
		Ile285	3.95			
Acetyl-morin	Hydrophobic interactions	Thr42	3.48			
		Trp104	3.24			
		Thr138	3.78			
		Ala141	3.00			
		Val154	3.36			
		Ile285	3.34			
	Hydrogen bonds	Gln157		3.31	×	✓

Table S4: Molecular interactions between morin and acetylated morin within the active site of TLL. Distance interactions between carbon atoms and distance between donor and acceptor atoms (hydrogen bonds) are displayed in the 4th and 5th row respectively.

Compound	Type of interaction	AA	Distance (Å)	Distance D-A (Å)	Protein donor	Side Chain
Morin	Hydrophobic interactions	Tyr21	3.78			
		Leu259	3.72			
	Hydrogen bonds	Arg84		3.74	✓	✓
		His258		3.26	×	✓
		Gly266		3.91	×	×
	π – stacking (T-type)	Trp89	4.88			
acetyl-morin	Hydrophobic interactions	Val203	3.71			
		Leu206	3.86			
		Leu259	3.87			
	Hydrogen bonds	Asn92		2.96	✓	✓
	Salt bridges	Arg84	4.44			
